# Research Trends and Mechanistic Insights Into Natural Medicines for Alzheimer's Disease: A Bibliometric Analysis and Narrative Review

**DOI:** 10.1002/ptr.70445

**Published:** 2026-09-11

**Authors:** Maoning Wang, Xiaolian Zhang, Huaxian Yang, Jianhe Zhao, Wentao Wei, Qian Zhao, Wenjia Wang, Yunhui Hu, Jiansong Fang

**Affiliations:** ^1^ State Key Laboratory of Traditional Chinese Medicine Syndrome, Science and Technology Innovation Center Guangzhou University of Chinese Medicine Guangzhou China; ^2^ Tianjin Tasly Digital Intelligence Chinese Medicine Technology Co., Ltd. Tianjin China

**Keywords:** alzheimer's disease, bibliometric analysis, gut microbiota, molecular docking, natural medicines, network pharmacology, pharmacological mechanisms

## Abstract

Natural medicines have been widely investigated for Alzheimer's disease (AD), but the development of this field and the relationship between bibliometric themes and experimental evidence remain insufficiently characterized. This study aimed to map the research landscape and thematic evolution of natural medicine research for AD through bibliometric analysis and to further examine representative mechanistic evidence through a focused narrative synthesis. MethodsPublications in the Web of Science Core Collection from January 1, 2001, to April 28, 2025 were analyzed using VOSviewer and CiteSpace. Bibliometric findings were used to guide a focused narrative synthesis of original experimental studies. A total of 3316 publications involving 16,858 authors, 3594 institutions, and 106 countries or regions were identified, with China contributing the most publications. Research has shifted from acetylcholinesterase inhibition and antioxidant effects toward broader AD‐related mechanisms, while computational approaches such as network pharmacology and molecular docking have become increasingly represented in this research field. Gut microbiota‐related research has increased but remains an emerging direction rather than a dominant hotspot. Experimental studies of representative compounds, medicinal herbs, and formulae mainly involved A*β* and tau pathology, cholinergic dysfunction, oxidative stress, neuroinflammation, mitochondrial dysfunction, and autophagy. Clinical evidence varied substantially, with EGb 761 and Huperzine A being more extensively studied than many agents supported mainly by preclinical evidence. Computational approaches were increasingly used for hypothesis generation, but their predictions require experimental validation. Natural medicine research for AD has become increasingly mechanism‐oriented and methodologically diverse. Future studies should strengthen experimental validation, improve standardization and pharmacokinetic characterization, and conduct adequately powered clinical trials to support translation of preclinical findings.

## Graphical abstract

Research landscape and mechanistic insights into natural medicines for Alzheimer's disease.

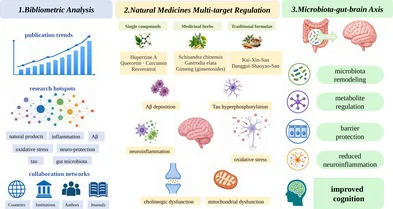

**Abbreviations**
ACh: Acetylcholine
AD: Alzheimer's disease
Akt: Protein kinase B
APP: Amyloid precursor protein
APP/PS1: Amyloid precursor protein/presenilin 1
A*β*
: Amyloid beta
BACE1: Beta‐site amyloid precursor protein‐cleaving enzyme 1
DSS: Danggui Shaoyao San
GSK‐3*β*
: Glycogen synthase kinase 3 beta
IF: Impact factor
IL‐1*β*
: Interleukin‐1 beta
NLRP3: NOD‐like receptor family pyrin domain‐containing 3
NMs: Natural medicines
Nrf2: Nuclear factor erythroid 2‐related factor 2
PC12: rat pheochromocytoma cell line
ROS: Reactive oxygen species
SIRT1: Sirtuin 1
TCM: Traditional Chinese medicine
TNF‐*α*
: Tumor necrosis factor alpha

## 1 Introduction

Alzheimer's disease (AD) is a neurodegenerative disorder characterized primarily by progressive cognitive decline and behavioral abnormalities. It predominantly affects the elderly population and has emerged as a major global public health challenge (Livingston et al. 2024). Epidemiological studies indicate that the prevalence of AD increases significantly with age (Scheltens et al. 2021). The pathogenesis of AD involves multiple complex pathways: the deposition of amyloid‐*β* (A*β*) in the brain is not only a key initiating pathological factor and a potential therapeutic target (Hua and Zhao 2023), but also prompts a shift in research focus towards tau protein, the primary component of neurofibrillary tangles, which constitute another core pathological hallmark (Congdon et al. 2023). Furthermore, genetic background, metabolic syndrome, and vascular factors also play significant roles in the onset and progression of the disease (Knopman et al. 2021; Scheltens et al. 2021).

Current pharmacological treatments for AD include cholinesterase inhibitors, the NMDA receptor antagonist memantine, and recently approved anti‐A*β* monoclonal antibodies. Lecanemab and donanemab have received FDA approval for the treatment of early AD based on clinical evidence from Phase 3 trials, including the Clarity AD and TRAILBLAZER‐ALZ 2 studies. These trials demonstrated reductions in amyloid burden and modest slowing of cognitive and functional decline, supporting their clinical application. However, these therapies do not reverse disease progression and are associated with important safety and implementation considerations (Sims et al. 2023; van Dyck et al. 2023). Despite these advances, current treatments provide limited benefit for many patients and do not address the full range of pathological processes involved in AD. This has continued to drive interest in alternative or complementary therapeutic approaches, including natural medicines with multiple bioactive constituents and potentially broader pharmacological effects.

Natural medicines (NMs), derived from plants, minerals, and other natural sources, possess a rich history of therapeutic application across diverse medical traditions worldwide, notably in China, Japan, Korea, and India (Leonti and Verpoorte 2017). These resources represent an invaluable repository for modern drug discovery, particularly for complex diseases. AD, a multifaceted neurodegenerative disorder with limited treatment options, stands as a prime candidate for exploring the potential of NMs.

In this context, traditional Chinese medicine (TCM) is one natural medicine system that has historically been used in the management of age‐related cognitive decline. Modern studies are increasingly exploring its potential mechanisms in AD. Traditional theories, such as “the kidney essence generating marrow” and “the brain being the sea of marrow,” have been used to interpret cognitive impairment within the TCM framework. Corresponding herbal formulas emphasize holistic regulation and syndrome differentiation. Recent studies suggest that TCM formulas may exert effects through multiple components, targets, and pathways, including modulation of A*β* pathology, tau phosphorylation, neuroinflammation, synaptic plasticity, cerebral blood flow, and energy metabolism. These findings suggest that natural medicines may offer potential value for AD‐related research and therapeutic development.

In recent years, research on natural medicines for the prevention and treatment of AD has increased substantially, generating a growing body of basic and clinical literature. However, the relevant studies are dispersed across multiple sources and cover a wide range of research topics, making it difficult for conventional narrative reviews to fully capture the overall knowledge structure and temporal evolution of the field. Bibliometric analysis, using visualization tools such as CiteSpace and VOSviewer, enables quantitative assessment of publication trends, collaboration networks, co‐citation relationships, keyword clusters, and emerging research topics. Nevertheless, bibliometric analysis primarily reflects the structure and development of a research field and cannot, by itself, establish the pharmacological efficacy of individual natural medicines or causal relationships between specific medicines and molecular mechanisms. Therefore, in the present study, bibliometric analysis was first used to characterize the research landscape and identify major thematic clusters, research hotspots, and representative natural medicines in the literature retrieved from January 1, 2001, to April 28, 2025. These findings were subsequently used to guide a focused narrative synthesis of relevant experimental studies. Importantly, the mechanistic evidence discussed in the subsequent sections was derived from original pharmacological studies rather than directly inferred from bibliometric co‐occurrence, clustering, or citation patterns. Through this integrated approach, we aimed to characterize the development of research on natural medicines for AD, identify major research themes and emerging directions, and further summarize and discuss the mechanisms of representative natural medicines based on available experimental evidence.

## 2 Materials and Methods

### 2.1 Literature Sources and Retrieval Strategy

Literature retrieval was conducted using the Web of Science Core Collection database, covering the period from January 1, 2001, to April 28, 2025. Because publication data for 2025 were incomplete at the time of retrieval, the annual publication trend was presented only for the complete calendar years from 2001 to 2024. Nevertheless, all eligible records retrieved through April 28, 2025 were retained in the bibliometric dataset and included in the subsequent analyses.

An initial Web of Science Core Collection search retrieved 4697 records, exported in tab‐delimited format and assigned unique IDs. DeepSeek assisted preliminary screening by identifying records unrelated to natural medicines for AD treatment based on titles, keywords, and abstracts. The tool returned only candidate exclusion IDs, which were then manually reviewed. All final inclusion and exclusion decisions were made by human assessment; no record was excluded solely on AI output.

The specific search strategy was as follows: (“Natural medicine” OR “Natural drug” OR “Traditional Chinese medicine” OR “Chinese medicine” OR “Chinese medicinal formula” OR “Prescription of traditional Chinese medicine” OR “Herb” OR “Medicinal plant” OR “Herbal medicine” OR “Plant extract” OR “Herb extract” OR “Herbal compound” OR “Plant compound” OR “Phytochemical”) AND (TS = (“Alzheimer” OR “Alzheimer disease” OR “Alzheimer's disease” OR “Senile Dementia”)). The detailed literature screening process is illustrated in Figure 1.

**FIGURE 1:**
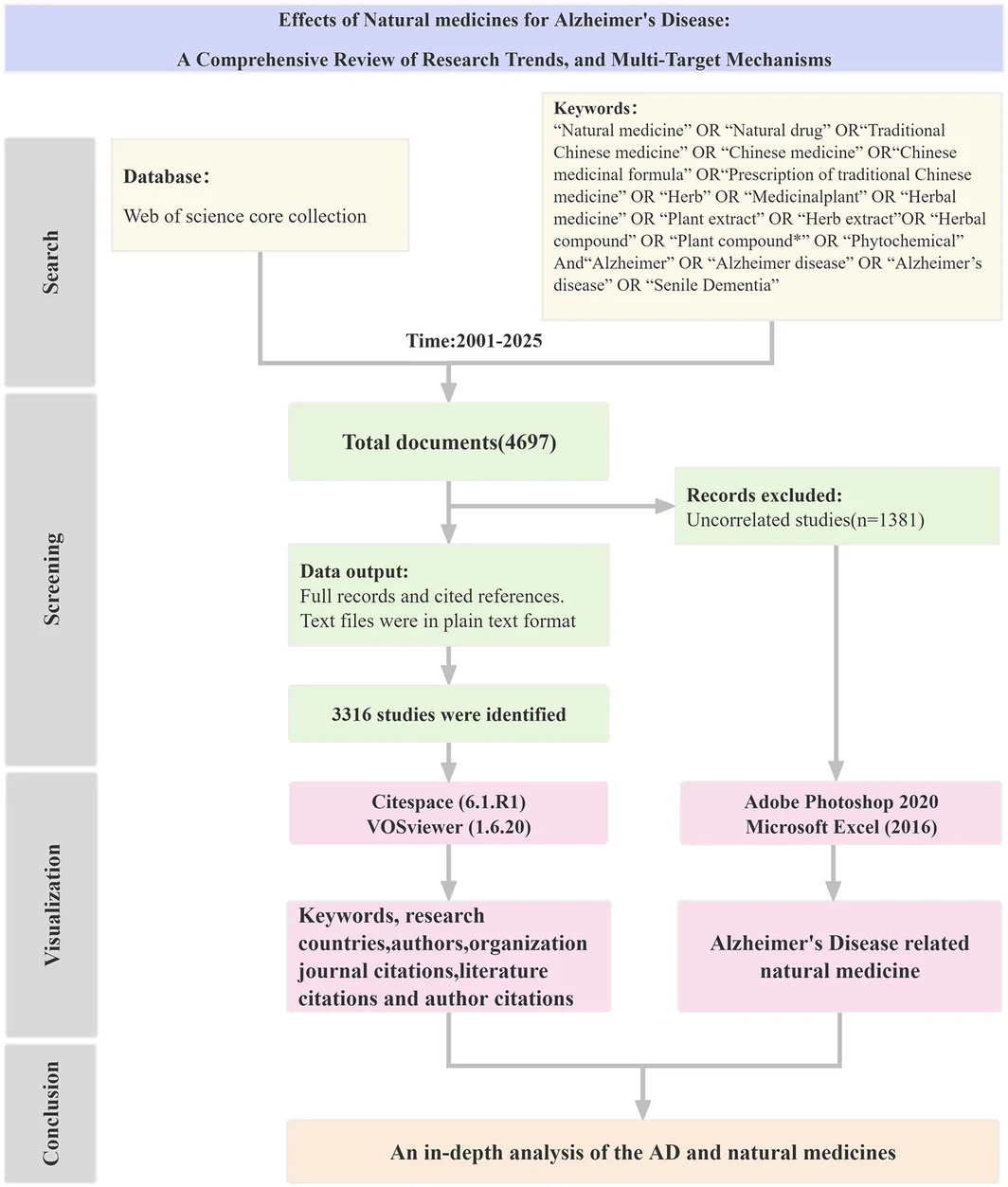
Flowchart of the inclusion and exclusion criteria.

### 2.2 Data Collection and Analytical Methods

Data analysis and visualization were performed using Microsoft Excel (2016), Adobe Photoshop 2020, VOSviewer 1.6.20, and CiteSpace 6.3.R1. Excel was employed for data collection, table generation, and analysis of annual publication trends. The software processed raw data to extract information including the number of publications per year, countries or regions, journals, document titles, and keywords. Adobe Photoshop 2020 was used for image integration. VOSviewer was utilized to visualize collaboration networks among countries, institutions, and authors, as well as co‐citation networks of journals, authors, and documents. For the collaboration networks, the minimum number of publications was set at 9 for authors, 16 for institutions, and 9 for countries. For the co‐citation analyses, the minimum number of citations was set at 41 for references, 682 for journals, and 63 for co‐cited authors. In these visualizations, node sizes represent the number of published articles, links indicate collaborative relationships, and link thickness reflects the strength of collaboration. Different colors represent distinct research clusters. The total link strength in the collaboration network measures the frequency of co‐authorship, highlighting the extent of collaborative research (Liu et al. 2024).

Keyword clustering analysis was performed using CiteSpace, with the *K* value set to 10. The co‐occurrence network was constructed using the Pathfinder algorithm and pruning the merged network (Sabe et al. 2022). A centrality value greater than 0.1 indicated strong correlations, accurately reflecting the specific parameter configurations of each node. For keyword cluster modularity analysis, the log‐likelihood ratio algorithm with *p* level was applied to categorize keywords into clusters. A *Q* value (modularity) above 0.3 signified a significant cluster structure, while an *S* value (silhouette score) above 0.5 indicated reasonable cluster cohesion, thereby validating the robustness of the clustering results.

### 2.3 Mechanistic Literature Synthesis

The mechanistic section was conducted as a focused narrative synthesis rather than a systematic review. Its structure was guided by the main mechanistic themes identified from the bibliometric analyses, particularly the keyword co‐occurrence and clustering results. These themes were used to organize the subsequent discussion of representative natural medicines and their potential mechanisms in AD.

To make the literature selection process more transparent, we further searched for original experimental studies related to the major mechanistic themes identified in the bibliometric analysis. Studies were selected if they provided direct experimental evidence for AD‐related mechanisms, with greater emphasis placed on in vivo studies and on studies reporting clear molecular or cellular outcomes. In vitro studies were also included when they provided relevant mechanistic evidence. Studies based only on network pharmacology, molecular docking, or other computational predictions without experimental validation were not used as the main evidence for specific mechanistic conclusions. Review articles were mainly used to provide background information and to help identify relevant original studies.

Representative natural medicines were selected by considering their occurrence in the bibliometric results, the diversity of medicine types, and the AD‐related pathological processes they represent. As summarized in Table S4, the selected medicines included single compounds, medicinal herbs, and traditional formulae and were intended as representative examples rather than an exhaustive list of all natural medicines identified in the bibliometric analysis.

The mechanistic discussion therefore reflects evidence from the original experimental literature, while the bibliometric results were used mainly to define the major themes and overall structure of the narrative synthesis.

## 3 Results

### 3.1 Trends of Annual Publications

Through the Web of Science Core Collection database, a thematic search was conducted for literature on natural medicine and AD published from January 1, 2001, to April 28, 2025. After the exclusion of off‐topic publications, a total of 3316 documents were ultimately curated for analysis.

Based on the examination of 3316 ad‐related publications, this study reveals the research output characteristics in the field. The annual publication volume is shown in Figure 2A. From 2001 to 2024, the number of publications in this category increased markedly from 10 to 361, with an average annual growth rate of 16.3%. Despite a transient decline in 2023 (a year‐on‐year decrease of 4.3%), publications rebounded rapidly in 2024 with a growth rate of 16.1%, reaching a record high and demonstrating continuously strengthening research vitality in the field. In terms of national contributions, as illustrated in Figure 2B, China secured the leading position globally with 1395 publications, significantly outpacing India (363 publications) and the United States (279 publications).

**FIGURE 2:**
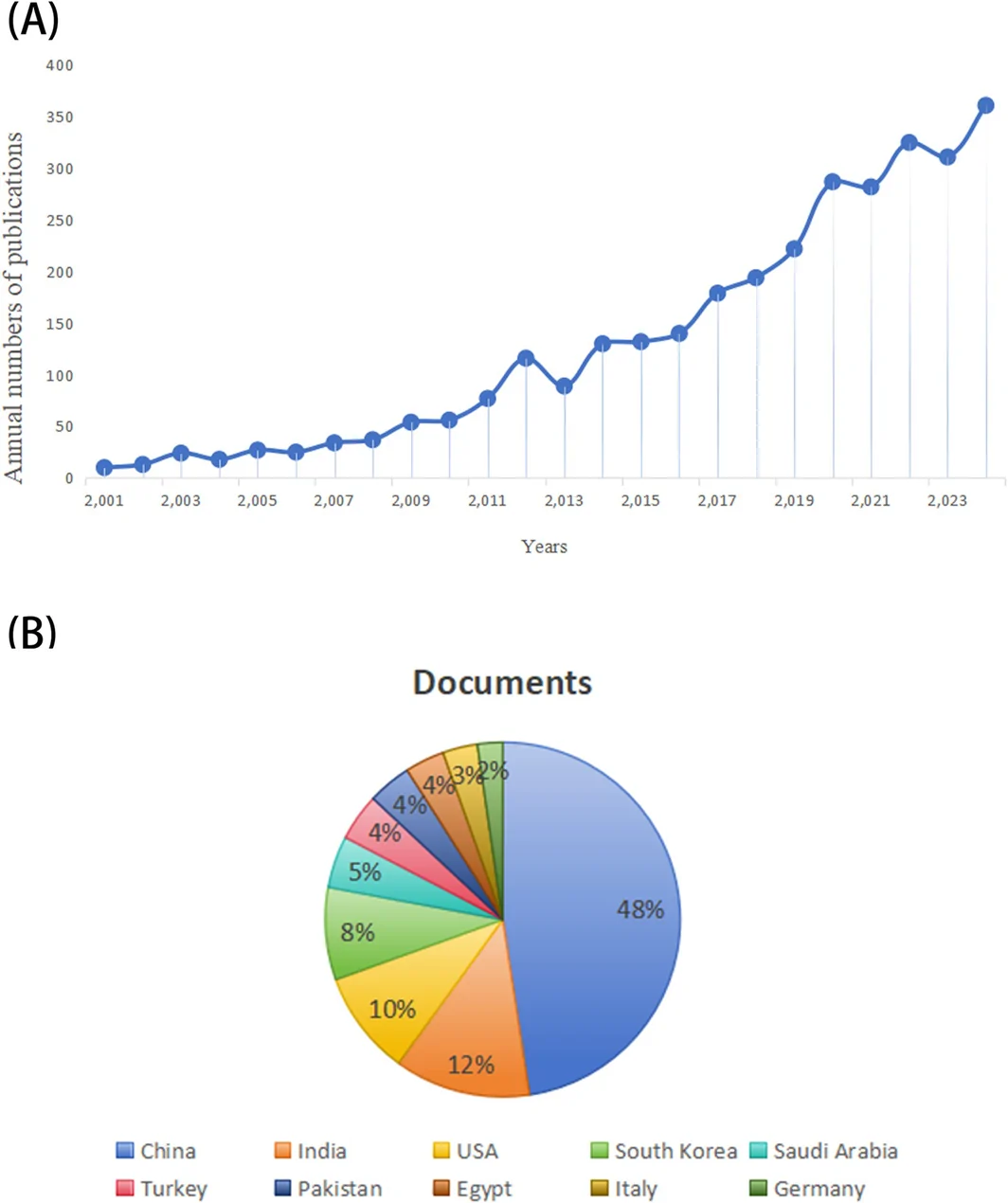
Annual publication trend and country distribution of publications on NMs against AD. (A) Annual publication trend from 2001 to 2024. (B) Distribution of publications by country/region based on all eligible records retrieved through April 28, 2025.

### 3.2 Cooperation Networks Between Countries, Institutions, and Authors

As shown in Figure 3A, in the author collaboration analysis, 16,858 authors were initially identified. After applying a minimum publication threshold of 9 articles, 74 authors were selected for collaboration network mapping. An in‐depth analysis of the top 10 authors by centrality strength revealed Turkey's Zengin Gokhan as the most productive contributor with 51 publications. Seven Chinese authors were prominently represented, with total link strengths of 34 (Shi, Jing), 33 (Bi, Kaishun; Tian, Jinzhou), 32 (Jia, Ying), 28 (Yan, Tingxu), 27 (Wei, Mingqing), and 26 (Wu, Bo) respectively, demonstrating their central positions in the collaboration network (Table S1).

**FIGURE 3:**
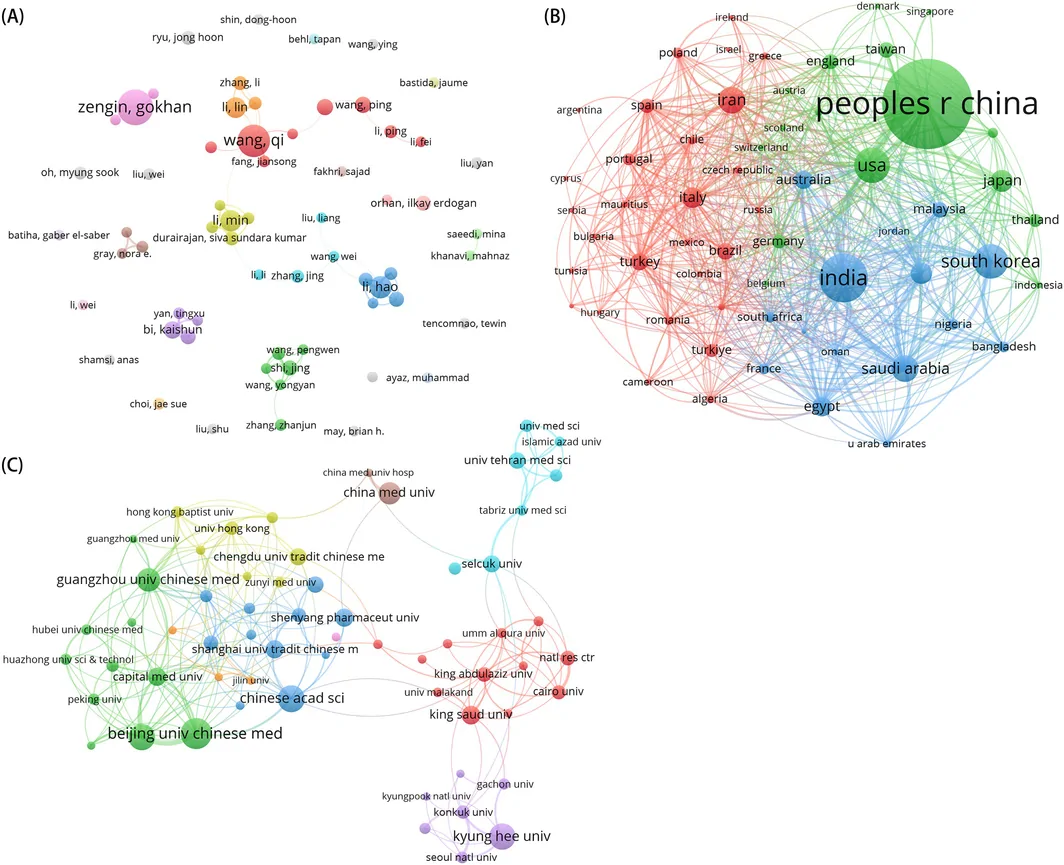
Knowledge maps of co‐authorship in the field of NMs for AD. (A) Author co‐authorship network, (B) country Co‐authorship network, and (C) Organization co‐authorship network. Different colors represent different clusters; the node size indicates the number of publications, and the line thickness represents the link strength between nodes.

In country/region collaboration analysis (Figure 3B), co‐occurrence examination identified 106 countries/regions. After implementing a minimum publication threshold of 9 articles, 54 countries were included for further analysis. Subsequent investigation showed that although the United States ranked third in total publication volume, it achieved the highest centrality (total link strength 281), indicating its pivotal role in international collaboration networks and significant academic influence as a bridging hub (Table S2).

For institutional co‐occurrence analysis (Figure 3C), 3594 institutions were initially identified. After applying a minimum publication threshold of 16 articles, 63 core institutions were selected for collaboration network mapping. Analysis of the top 10 institutions by centrality strength (Table 1) revealed China's most active institutions including China Academy of Chinese Medical Sciences (63 publications/907 citations), Beijing University of Chinese Medicine (81 publications/919 citations), Chinese Academy of Sciences (81 publications/2154 citations), and Guangzhou University of Chinese Medicine (58 publications/1124 citations). China Academy of Chinese Medical Sciences maintained the most extensive collaboration network (Total Link Strength = 59), followed by Beijing University of Chinese Medicine and Chinese Academy of Sciences. Among the top 10 collaborative institutions, Selcuk University (Ave = 32.5) and Chinese Academy of Medical Sciences (Ave = 29.4) demonstrated higher average citation counts per publication compared to other institutions.

**TABLE 1:** The top 10 cooperative institutions regarding natural medicines against AD from 2001 to April 2025.

Rank	Institutions	Total link strength	Documents	Citations	Average citations
1	Beijing Univ Chinese Med	58	81	919	11.3
2	Chinese Acad Sci	55	81	2154	26.6
3	China Acad Chinese Med Sci	59	63	907	14.4
4	Guangzhou Univ Chinese Med	45	58	1124	19.4
5	Selcuk Univ	37	57	1855	32.5
6	Capital Med Univ	43	43	855	19.9
7	China Med Univ	30	42	661	15.7
8	Chinese Acad Med Sci	31	34	998	29.4
9	Jinan Univ	34	28	571	20.4
10	China Med Univ Hosp	30	20	299	14.9

*Note:* TLS (total link strength) represents the sum of the collaboration link strengths between a given institution and all other institutions in the VOSviewer co‐authorship network. Higher TLS values indicate stronger overall collaborative connections in this field.

### 3.3 Citation/Co‐Citation Analysis of Authors, Journals, and References

Co‐citation analysis serves as a metric for article merit evaluation. Co‐cited authors refer to scholars whose research outputs are simultaneously cited by multiple publications. The visualized network map of author co‐citation is shown in Figure 4A. A total of 77,031 authors were screened. Setting the minimum citation count to 63 for authors, 96 authors met this threshold. Ellman, G.L. ranked first with 239 co‐citation counts and a Total Link Strength (TLS) of 1249, indicating his significant influence and extensive collaborative network within this field. Selkoe, D.J. and Butterfield, D.A. ranked second and third, respectively (Table S3).

**FIGURE 4:**
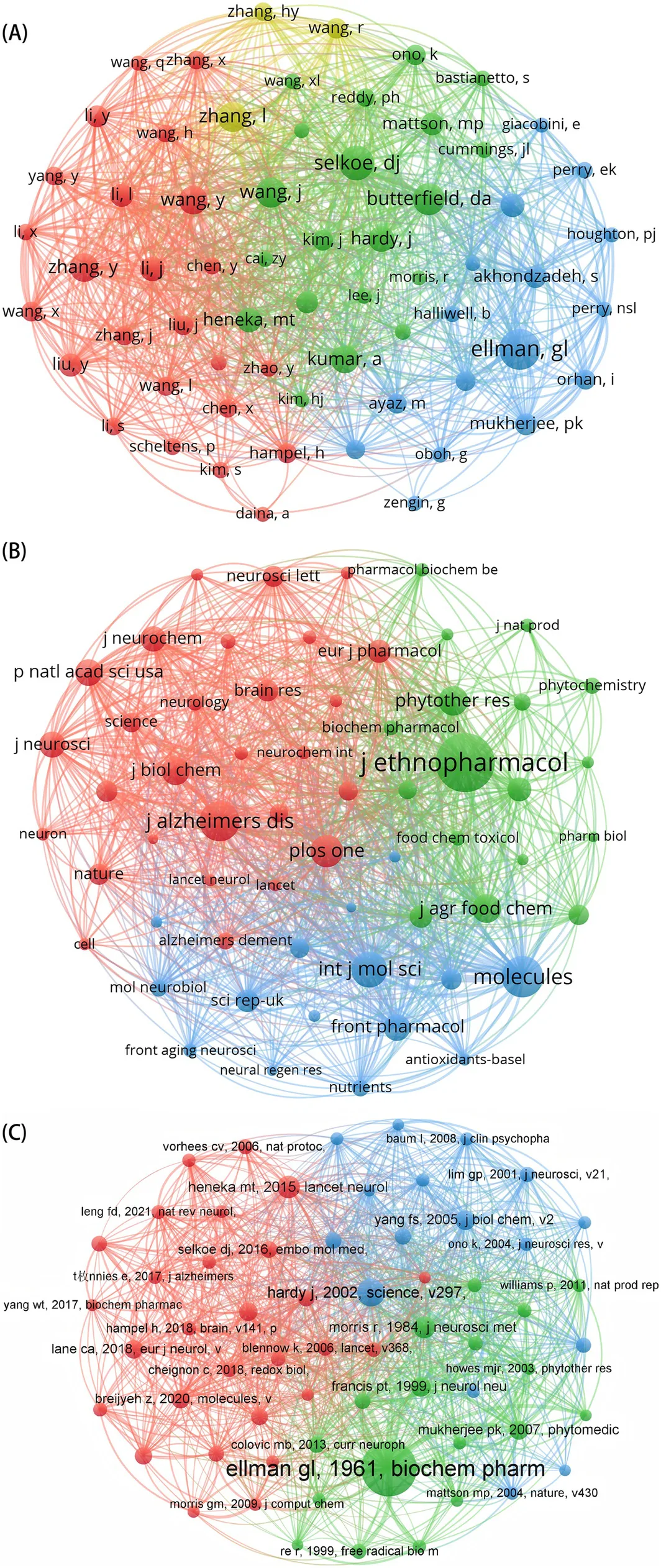
Knowledge maps of co‐citation analysis for publications on NMs against AD, including (A) Co‐citation of authors, (B) Co‐citation of journals, and (C) Co‐citation of references. Different colors indicate different clusters; node size represents the number of citations, and the distance between nodes indicates their correlation.

G.L. Ellman, from Dow Chemical's Biochemical Research Laboratory in Michigan, USA, conducted cross‐disciplinary research and developed the Ellman method for acetylcholinesterase activity measurement. This approach uses acetylthiocholine hydrolysis (catalyzed by the enzyme) and DTNB reaction to form a yellow compound absorbable at 412 nm, enabling rapid, accurate testing with simplicity, high sensitivity, and good reproducibility, supporting AD‐related research. Selkoe D.J. from Harvard Medical School, a multiple‐time Highly Cited Researcher in Neuroscience and Behavior, established the dominant amyloid hypothesis (reshaping AD understanding), elucidated A*β*'s aggregation from soluble synaptotoxic oligomers to insoluble plaques, clarified A*β* (upstream trigger) and Tau tangles (downstream executor) causal link, laid foundations for AD biomarker diagnosis (e.g., CSF A*β*/Tau tests) and A*β*‐targeted drugs (e.g., immunotherapies), and is a key founder of modern AD research paradigms.

In the journal co‐citation analysis (Figure 4B), a total of 3316 publications related to natural medicines for AD retrieved from January 1, 2001, to April 28, 2025 were analyzed, resulting in 19,053 records. With a minimum citation threshold set at 682, a total of 61 journals met this criterion. The top 10 most cited journals identified by VOSviewer are presented in Table 2. The Journal of Ethnopharmacology ranked first, with 144 relevant publications and a total of 3753 citations during this period, followed by Molecules (2397 citations), the Journal of Alzheimer's Disease (1891 citations), PLOS ONE (1751 citations), and the Journal of Biological Chemistry (1749 citations). The Journal of Biological Chemistry (Impact Factor (IF) = 3.9), Proceedings of the National Academy of Sciences of the United States of America (IF = 9.1), and the Journal of Neuroscience Research (IF = 3.4) exhibited the highest average citations per paper, indicating a strong per‐article impact of the relevant studies published in these journals. Notably, six of the top 10 journals were categorized as Q1 or Q2 in the Journal Citation Reports (JCR), underscoring their significant academic influence. It is also noteworthy that these high‐impact journals were predominantly based in the USA and Europe, highlighting the dominant role of Euro‐American countries in publishing influential research in this field.

**TABLE 2:** The top 10 most active journals in research of natural medicines against AD (sorted by total citation) from 2001 to April 2025.

Rank	Journal	Total citations	Documents	Average citations per papers	IF(2024)	JCR	Country
1	Journal of Ethnopharmacology	3753	144	26.1	5.4	Q1	Netherlands
2	Molecules	2397	89	26.9	4.6	Q2	Switzerland
3	Journal of alzheimers disease	1891	46	41.1	3.1	Q2	Netherlands
4	PLOS ONE	1751	13	134.7	2.6	Q2	USA
5	Indian Journal of Pharmaceutical Sciences	1603	7	229	/	Q4	India
6	Journal of Biological Chemistry	1749	1	1749	3.9	Q2	USA
7	Journal of Food Biochemistry	1753	20	87.7	4.2	Q2	USA
8	Journal of Neuroscience Research	1541	6	256.8	3.4	Q2	USA
9	Proceedings of the National Academy of Sciences of the United States of America	1593	1	1593	9.1	Q1	USA
10	Frontiers in Pharmacology	1235	131	9.4	4.8	Q1	Switzerland

*Note:* JCR refers to the Journal Citation Reports quartile ranking of a journal within its subject category. Q1 denotes the highest ranked quartile, followed by Q2, Q3, and Q4.

In the reference co‐citation analysis, a total of 123,411 references were screened. When the minimum citation threshold of 41 was set, 62 references met this criterion (Figure 4C). Table 3 lists the top 10 most cited references, with the top three most cited references as follows. Developed by Ellman, G.L. et al. this work introduced an innovative improvement to the method for measuring acetylcholinesterase activity (Ellman et al. 1961). It presented a rapid and sensitive colorimetric assay, providing a standardized tool for solving key enzyme activity measurement challenges in neurophysiology and toxicology.

**TABLE 3:** Top 10 highest cited references in natural medicines against AD from 2001 to April 2025.

No.	Title	Journal	Total citations	IF	Publication year	First author
1	A new and rapid colorimetric determination of acetylcholinesterase activity	Biochem Pharmacol	485	5.6	1961	ELLMAN GL
2	Antioxidant activity applying an improved ABTS radical cation decolorization assay	Free Radical Biology and Medicine	93	8.2	1999	Re R
3	Acetylcholinesterase inhibitors from plants	Phytomedicine	86	8.3	2007	Mukherjee PK
4	The amyloid hypothesis of Alzheimer's disease: progress and problems on the road to therapeutics	Science	132	45.8	2002	Hardy J
5	Use of a free radical method to evaluate antioxidant activity	Lwt‐Food Science and Technology	56	6.6	1995	Brand Williams
6	The ferric reducing ability of plasma (FRAP) as a measure of “antioxidant power”: the FRAP assay	Analytical Biochemistry	58	2.5	1996	Benzie IF
7	Alzheimer's disease: Targeting the Cholinergic System	Curr Neuropharmacol	66	5.3	2016	Ferreira‐Vieira TH
8	Antioxidative Activities of Product of Browning Reaction Prepared from Glucosamine	Japan Journal of nutrition	59	/	1986	Oyaizu, M
9	The cholinergic hypothesis of Alzheimer's disease: a review of progress	Journal neurol neurosurg psychiatry	71	7.5	1999	Francis PT
10	Developments of a water‐maze procedure for studying spatial learning in the rat	Journal of Neuroscience Methods	112	2.3	1984	Morris R

Published by Hardy and Selkoe, this landmark paper provided an influential formulation of the amyloid hypothesis of AD (Hardy and Selkoe 2002). More recent clinical trials have provided further evidence for the therapeutic relevance of A*β* in early AD. The Phase 3 Clarity AD trial provided clinical evidence supporting the clinical approval of lecanemab by demonstrating reduced brain amyloid burden and moderately slower cognitive and functional decline compared with placebo over 18 months (van Dyck et al. 2023). Similarly, the TRAILBLAZER‐ALZ 2 trial provided clinical evidence supporting the approval of donanemab by demonstrating reduced amyloid burden and slowed clinical progression in patients with early symptomatic AD (Sims et al. 2023). These findings support A*β* as a clinically relevant therapeutic target while also indicating that amyloid reduction alone does not fully address the complex pathogenesis of AD.

Developed and validated by Morris, R.G.M., this study established a water maze procedure for studying spatial learning in rats (Morris 1984). The research established this paradigm as an effective tool for assessing spatial memory, demonstrating that rats can use external visual cues to locate a hidden platform in water, forming a “cognitive map.” It further revealed that this learning process is highly dependent on hippocampal function. This finding established the water maze as a core classical method in neuroscience for studying learning and memory mechanisms.

### 3.4 Co‐Occurrence Network Analysis of Keywords

Keywords serve as condensed summaries of literature content, and keyword co‐occurrence network analysis can reveal research hotspots in a specific field during a given period. In this study, the keyword co‐occurrence network and cluster map generated through CiteSpace analysis are presented in Figure 5A and Figure 5B, respectively. A centrality value greater than 0.1 indicates a strong correlation, suggesting that keywords such as acetylcholinesterase, acetylcholinesterase inhibitors, oxidative stress, antimicrobial activity, and cell death are closely associated with AD. The keywords were further categorized into 15 clusters (Figure 5B). The modularity (*Q* value) of the cluster map was 0.7623, exceeding the threshold of 0.3, which signifies a significant cluster structure. The mean silhouette (*S* value) was 0.9197, greater than 0.5, indicating that the clustering is reasonable and robust.

**FIGURE 5:**
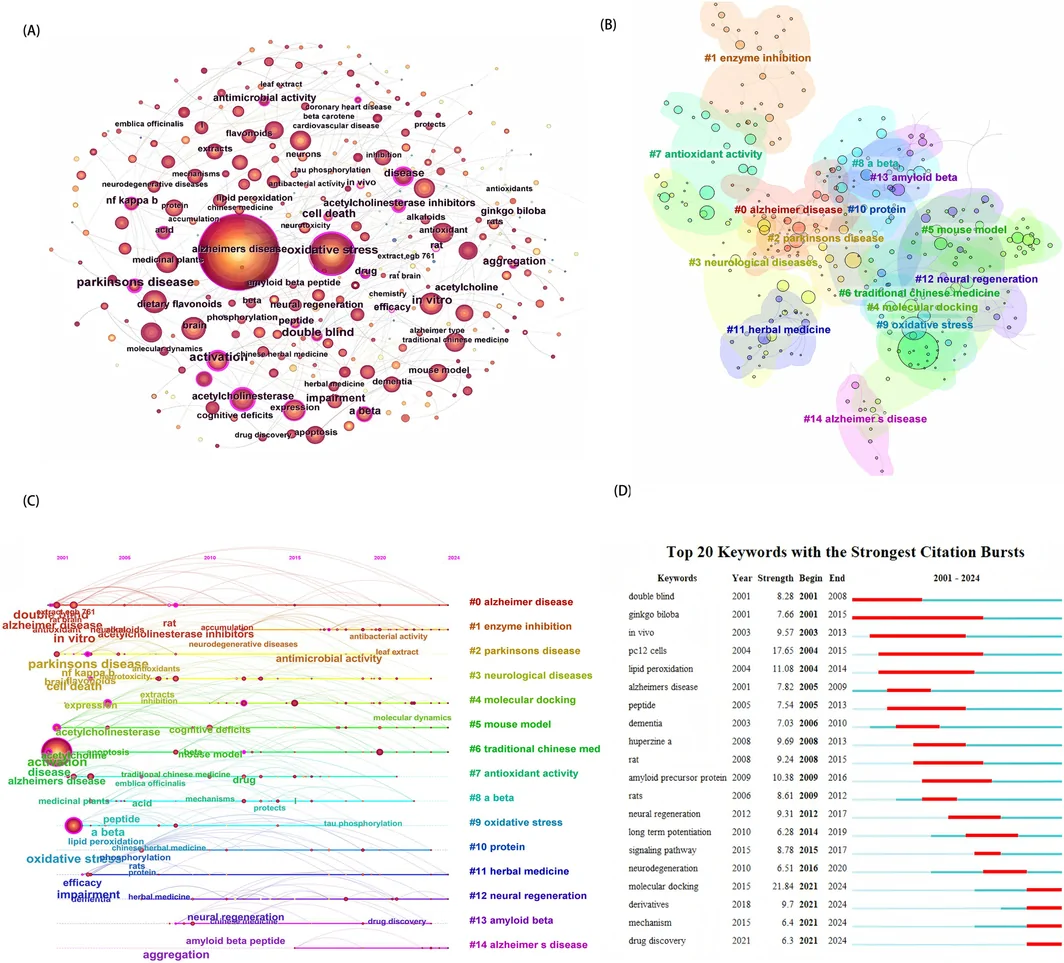
Visualization of keyword co‐occurrence and citation bursts for NMs against AD. (A) Network visualization, (B) Clustering module, and (C) Timeline view. (D) The top 20 keywords with the strongest citation bursts. Strength represents the intensity of the citation burst; Begin and End indicate the starting and ending years of the burst, respectively, and the red bars indicate the periods during which citation bursts were detected.

After excluding search terms and semantically ambiguous keywords, 15 keyword clusters were identified, and the leading keywords within each cluster are summarized in Table 4. The silhouette values of these clusters ranged from 0.889 to 1.000, indicating good internal consistency and reliability of the clustering results. Among them, the two largest clusters were Cluster 0, “Traditional Chinese medicine,” and Cluster 1, “Oxidative stress,” each containing 28 keywords. Cluster 0, featuring TCM, network pharmacology, and natural products, reflects the close association between traditional medicine‐related resources and methodological approaches in this research field and provided a reference for selecting representative medicines. Cluster 1 (“Oxidative stress”) highlighted oxidative stress, inflammation, and gut microbiota as interconnected themes.

**TABLE 4:** Table of keyword clusters and the top three keywords in each cluster.

ClusterID	Size	Silhouette	Label (LLR)	Top 3 items by frequency in the cluster
0	28	0.894	Traditional Chinese medicine	Traditional Chinese medicine; network pharmacology; natural products
1	28	0.947	Oxidative stress	Oxidative stress, inflammation, gut microbiota
2	24	0.941	A*β*	Amyloid beta, neuroinflammation, cells
3	22	0.917	Protein	Medicinal plants, protein, tau
4	20	0.934	Enzyme inhibition	Antioxidant activity, essential oil, acetylcholinesterase
5	20	0.889	Brain	Brain, neurotoxicity, parkinsons disease
6	19	0.954	In vitro	In vitro, antioxidant, acetylcholinesterase inhibitors
7	18	0.974	Molecular docking	Neurodegenerative diseases; molecular docking; inhibitors
8	18	0.894	Activation	Activation, apoptosis, stress
9	17	0.930	Leaves	Leaves, dietary flavonoids, cell death
10	15	0.919	Herbal medicine	Dementia, impairment, herbal medicine
11	14	0.908	Acid	Extract, mechanisms, extracts
12	11	1	S disease	Memory, nitric oxide, signaling pathway
13	10	1	*β*	Expression, damage, cognitive impairment
14	10	0.968	*caenorhabditis elegans*	Model, inhibition, alzheimers

*Note:* Size indicates the number of keywords included in each cluster. Silhouette indicates the internal consistency and reliability of each keyword cluster. *A* value closer to 1 suggests higher homogeneity and better clustering quality.

Clusters 2 (“A*β*”) and 3 (“Protein”) centered on amyloid beta, tau pathology, neuroinflammation, and cellular mechanisms. Clusters 4 (“Enzyme inhibition”) and 6 (“In vitro”) reflected attention to cholinergic dysfunction, antioxidant activity, and acetylcholinesterase inhibition. Cluster 7 (“Molecular docking”) indicated increasing use of computational approaches, while Cluster 8 (“Activation”) emphasized apoptosis and stress responses. Additional clusters covering herbal extracts, memory, and signaling pathways demonstrated expansion from general efficacy to mechanism‐oriented research. Overall, current AD natural medicine research focuses on TCM resources, A*β*/tau pathology, cholinergic dysfunction, oxidative stress, neuroinflammation, gut microbiota, apoptosis, and mechanism‐driven evaluation, providing a bibliometric foundation for the subsequent mechanistic review.

Keyword clusters were analyzed using the Timeline View to generate a chronological distribution map (Figure 5C), which visualizes the average publication year of keywords within each cluster. Clusters positioned toward the right side of the figure represent more recent publications, while those on the left correspond to research themes primarily published before or around 2010. Analysis of the timeline indicates that recent studies have focused on molecular dynamics, molecular dynamics simulation, receptor modeling, *α*‐amylase, bioavailability, and depression.

For timeline‐based hotspot detection, research hotspots over the years were identified using a minimum duration of 3 years and a *Y* value threshold of 1.9 (Figure 5D). The results showed that molecular docking has been a prominent research topic since 2021, while earlier bursts included studies on PC12 cells and lipid peroxidation beginning in 2004. More recent hotspots have focused on derivative formulations and drug development. Gut–brain axis‐related studies have also increased in recent years, with 2 publications in 2020, 2 in 2022, 9 in 2023, 7 in 2024, and 11 in 2025. However, gut–brain axis‐related terms were not included among the top 20 keywords with the strongest citation bursts, indicating that this topic remains a relatively emerging direction despite the recent increase in publication output.

## 4 Discussion

This study conducted a bibliometric analysis of literature related to natural medicine‐based therapies for AD retrieved from the Web of Science Core Collection between January 1, 2001, and April 28, 2025, using VOSviewer and CiteSpace. It systematically outlines the evolution and research landscape of the field. Through visualization, the study clearly presents annual publication trends, country/regional distribution, research institutions, core journals, influential authors, key references, and high‐frequency keywords. The aim is to help readers gain a comprehensive understanding of global research frontiers and evolving trends, and to provide valuable insights for researchers already working in or considering entering this field.

The Web of Science Core Collection was selected for its rigorous journal screening, international visibility, and standardized bibliographic records with complete citation data—features well‐suited for bibliometric analysis using VOSviewer and CiteSpace.

### 4.1 Research Overview and Characteristics of Publications

This study conducted a bibliometric and visual analysis of the literature related to natural medicines for the treatment of AD during the study period. The results reveal distinct phase characteristics in the development of this research field.

The period 2001–2011 represented the initial research stage, characterized by relatively slow growth in the number of publications, with the annual publication volume remaining at a low level. Starting from 2012, the field entered a rapid development phase, marked by a significant increase in publications and an average annual growth rate of 16.3%. Although a temporary decline occurred in 2023, the number rebounded rapidly in 2024, reaching a historical high (361 publications). This indicates sustained research interest in the field, which is likely to remain an active area of investigation.

Analysis of international collaboration shows that China holds a dominant position in terms of publication output (1395 publications), far surpassing India (363 publications) and the USA (279 publications). Although the USA ranks third in total publications, it exhibits the highest centrality in the international collaboration network, highlighting its pivotal role as an academic exchange hub. Highly influential authors, such as Zengin Gokhan (Turkey) and several scholars from China, are active within the collaboration networks, reflecting the research strength and close collaboration among Chinese scholars. However, core journals are still primarily based in the USA and Europe, such as the Journal of Ethnopharmacology, Molecules, and PLOS ONE, suggesting that European and American countries maintain an advantage in terms of academic influence.

Chinese institutions performed prominently in this research. Institutions such as the China Academy of Chinese Medical Sciences, Beijing University of Chinese Medicine, and the Chinese Academy of Sciences ranked at the top in terms of publication output, citation counts, and collaboration networks, demonstrating China's substantial accumulated expertise and institutional support in natural medicine research. This is closely related to the traditional theoretical foundation of Chinese medicine in AD prevention and treatment, coupled with modern research investment.

This study analyzed a total of 3316 publications, focusing on the 61 most cited journals. Among these, the Journal of Ethnopharmacology emerged as the core journal, leading with 144 relevant publications and 3753 citations. High‐impact journals were primarily concentrated in the fields of pharmacology, ethnopharmacology, and neuroscience, such as the Journal of Biological Chemistry and Proceedings of the National Academy of Sciences of the United States of America. The high average citation counts of these journals indicate the significant academic influence of the research they publish.

Taken together, the field of natural medicines for AD is in a phase of rapid development. Based on the current evidence, priority should now be given to promoting cross‐border and inter‐team collaboration and facilitating the translation of mechanistic findings into clinically relevant research.

### 4.2 Research Trends and Hot Spots on NMs Against AD

After characterizing the overall research landscape through bibliometric analysis, we further examined the keyword clusters to identify representative natural medicines and the main mechanistic themes in this field. The 15 clusters were grouped into three broader categories: traditional Chinese medicine and natural medicine resources, AD‐related pathological mechanisms, and methodological approaches for pharmacological research (Table 4). Clusters related to TCM, herbal medicine, and natural products showed that natural medicines represent an important component of the current research landscape. The representative medicines discussed below were therefore grouped into single compounds, herbal medicines, and formulae. Table S4 summarizes the occurrence frequencies of representative single compounds, medicinal herbs, and formulae identified within the “Traditional Chinese medicine” cluster. Rather than selecting examples solely on the basis of frequency, we considered their bibliometric occurrence, representation of different types of natural medicines, and coverage of different AD‐related pathological processes. Huperzine A, curcumin, quercetin, and resveratrol were included as representative compounds; Schisandra chinensis, Gastrodia elata, and ginseng and its major ginsenosides were included as representative medicinal herbs or bioactive constituents; and Kai‐Xin‐San and Danggui Shaoyao San were selected as representative formulae. These examples were chosen to cover several recurring mechanistic themes in the bibliometric analysis, including cholinergic dysfunction, A*β* and tau pathology, oxidative stress, neuroinflammation, and related signaling pathways. They are intended to illustrate the major directions of mechanistic research rather than to provide an exhaustive account of all natural medicines identified in the dataset.

Mechanism‐related clusters, together with high‐frequency terms such as oxidative stress, amyloid beta, inflammation, neuroinflammation, tau, and gut microbiota, indicated that A*β* and tau pathology, oxidative stress, neuroinflammation, and related pathological processes have been recurring themes in this field. Gut microbiota‐related research has also received increasing attention, although it remains an emerging direction. Methodology‐related clusters, including molecular docking, in vitro studies, and enzyme inhibition, further reflected the growing use of mechanism‐oriented research approaches. Taken together, these bibliometric findings were used to organize the following discussion of representative experimental studies and natural medicines.

### 4.3 Gut Microbiota‐Related Mechanisms in Natural Medicine Research for AD

Keyword clustering analysis showed that gut microbiota appeared as a high‐frequency term within the “Oxidative stress” cluster (Table 4). Although gut microbiota‐related studies have increased in recent years, the overall number of publications remains relatively limited, and gut–brain axis‐related terms were not included among the top 20 keywords with the strongest citation bursts. These results suggest growing interest in microbiota‐related mechanisms in natural medicine research for AD, while this remains an emerging area. The main mechanisms discussed in this field are summarized in Figure 6.

**FIGURE 6:**
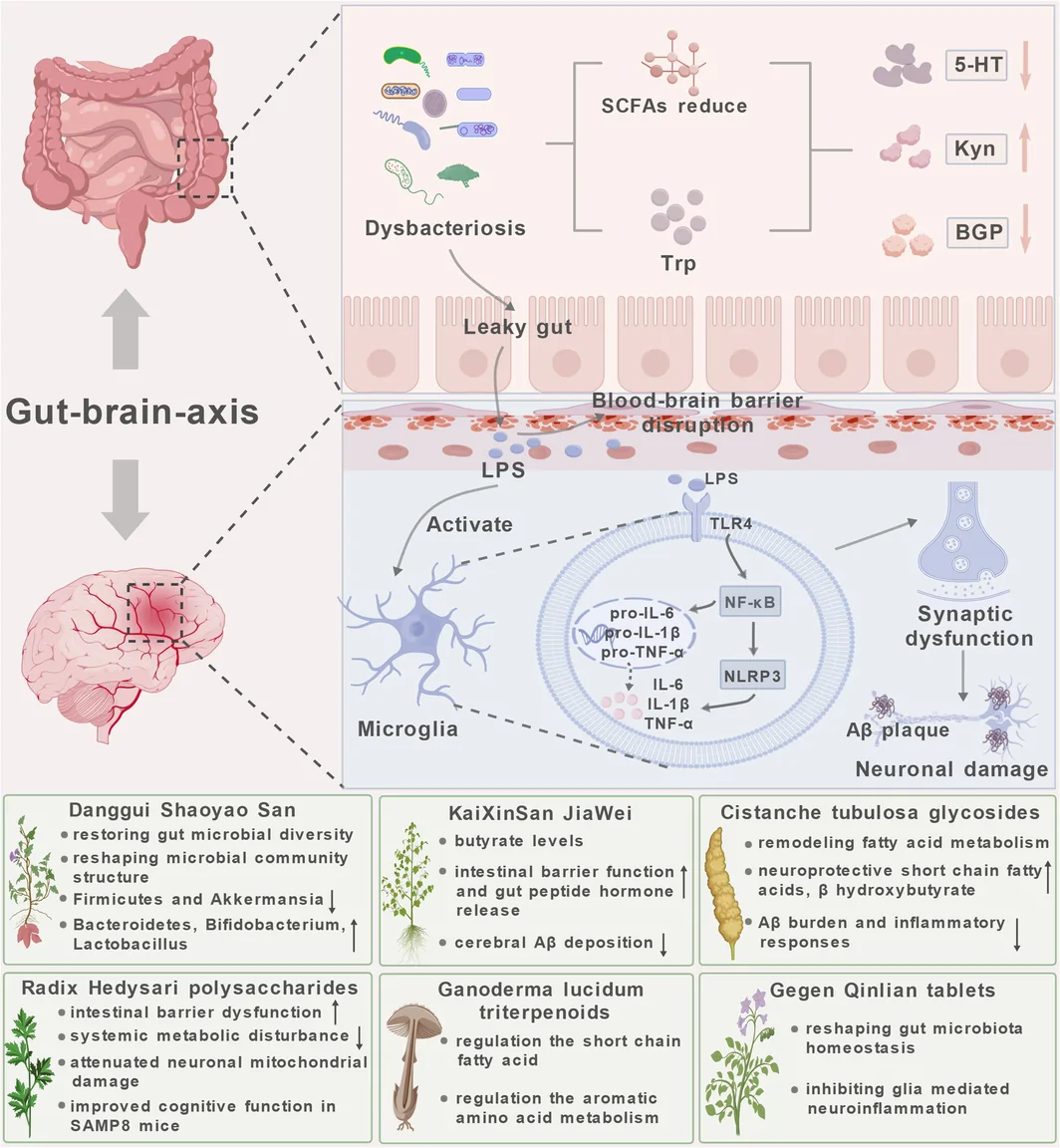
Gut–brain axis‐related mechanisms in AD and the effects of NMs. (Created with BioGDP.com).

Several experimental studies have examined these mechanisms in greater detail. By restoring gut microbial diversity and reshaping microbial community structure, Danggui Shaoyao San improved spatial memory impairment in APP/PS1 mice, reduced the abundance of taxa, including Firmicutes and Akkermansia, increased the abundance of Bacteroidetes, Bifidobacterium, and Lactobacillus, and further regulated D glutamine metabolism and bile acid biosynthesis (Liu et al. 2025). By modulating gut microorganisms and their metabolites, KaiXinSan JiaWei increased butyrate levels, improved intestinal barrier function and gut peptide hormone release, reduced cerebral amyloid deposition, and ultimately alleviated cognitive impairment in APP/PS1 mice (Mana et al. 2025). By enriching Akkermansia and remodeling fatty acid metabolism, Cistanche tubulosa glycosides increased the levels of neuroprotective short chain fatty acids such as *β* hydroxybutyrate, decreased proinflammatory long chain fatty acids in serum and brain tissue, and suppressed both A*β* burden and inflammatory responses, thereby revealing a gut microbiota, fatty acid metabolism, and neuroinflammation axis (Hou et al. 2025). By alleviating intestinal barrier dysfunction and correcting systemic metabolic disturbance, Radix Hedysari polysaccharides improved cognitive function in SAMP8 mice and attenuated neuronal mitochondrial damage (Yang et al. 2025). In addition, Ganoderma lucidum triterpenoids have been associated with the regulation of short chain fatty acids and aromatic amino acid metabolism, whereas Gegen Qinlian tablets were reported to delay AD progression by reshaping gut microbiota homeostasis and inhibiting glia mediated neuroinflammation (Shen et al. 2024; Wang et al. 2024). Collectively, natural medicines may not only reshape gut microbiota composition, but also exert therapeutic effects through metabolite reprogramming, barrier protection, suppression of immune inflammation, and the downstream attenuation of amyloid pathology and cognitive decline.

### 4.4 Common NMs Used in AD

The “Traditional Chinese medicine” cluster contained a number of frequently occurring terms related to single compounds, medicinal herbs, and traditional formulae (Table S4). For the following mechanistic discussion, representative medicines were selected by considering how often they appeared in the bibliometric analysis and whether they represented different types of natural medicines and AD‐related pathological processes. Based on these considerations, Huperzine A, quercetin, curcumin, and resveratrol were selected as representative compounds; Schisandra chinensis, Gastrodia elata, and ginseng, with particular attention to its major active constituents, ginsenosides, were selected as representative medicinal herbs; and Kai‐Xin‐San and Danggui Shaoyao San were included as representative formulae. These examples were not intended to cover all natural medicines identified in the bibliometric dataset, but to provide representative examples of the main mechanistic themes observed in this field.

The selected medicines differ in their main pharmacological features and together cover a broad range of AD‐related pathological processes (Table 5). Among the representative compounds, Huperzine A is mainly associated with cholinergic regulation, A*β* and tau pathology, and mitochondrial apoptosis, whereas quercetin, curcumin, and resveratrol have been studied more extensively in relation to A*β*‐associated changes, oxidative stress, neuroinflammation, autophagy, and mitochondrial dysfunction. Gastrodia elata and Schisandra chinensis show broader effects involving oxidative stress, neurotransmitter regulation, A*β*‐related pathology, inflammatory responses, and other neuroprotective pathways. For ginseng, much of the experimental evidence has focused on ginsenosides, which have been linked to A*β* and tau pathology, neurotransmitter dysfunction, mitochondrial impairment, oxidative stress, and neuroinflammation. The two representative formulae, Danggui Shaoyao San and Kai‐Xin‐San, further illustrate the multi‐component nature of traditional Chinese medicine, with reported effects on several pathological processes including A*β* metabolism, synaptic dysfunction, mitochondrial homeostasis, neuroinflammation, and gut microbiota‐related changes (Figure 7). The following sections discuss the experimental evidence for these representative medicines in greater detail.

**TABLE 5:** Representative natural medicines/formulae for Alzheimer's disease and their key pathways/targets and corresponding pathological processes.

Natural medicine/formula	Key pathways/targets	Corresponding pathological processes in AD
Huperzine A	AChE inhibition; GSK‐3*β*; p53‐related apoptotic signaling; mitochondrial protective signaling	Cholinergic dysfunction; A*β* aggregation and toxicity; tau hyperphosphorylation; mitochondrial dysfunction; neuronal apoptosis
Quercetin	Inhibition/disaggregation of A*β* fibrils; antioxidant defense; A*β* clearance; modulation of astrocytic A*β* uptake	A*β* aggregation and clearance; oxidative stress; astrogliosis; synaptic dysfunction
Curcumin	PI3K/Akt/mTOR; autophagy; autophagosome‐lysosome fusion; retrograde axonal transport	A*β* accumulation; oxidative and inflammatory injury; impaired autophagic clearance
Resveratrol	SIRT1/ROCK1; mitophagy (Parkin, Beclin‐1, LC3); Ca^2+^ homeostasis	A*β*‐induced neuronal injury; oxidative stress; mitochondrial dysfunction; apoptosis
*Schisandra chinensis*	Cholinergic signaling; GSK‐3*β*; PI3K/Akt/CREB; NF‐κB/NLRP3; SIRT1‐PGC‐1*α*‐Tau; BDNF/ERK/CREB; gut microbiota/SCFAs	A*β* deposition; tau hyperphosphorylation; neuroinflammation; oxidative stress; cholinergic dysfunction; gut microbiota and metabolic dysregulation
*Gastrodia elata* Blume	Nrf2/HO‐1; PKR/eIF2*α*/BACE1; DAF‐16/FOXO; cholinergic modulation	Oxidative stress; A*β*‐induced neurotoxicity; A*β* production and deposition; neurotransmitter/cholinergic dysfunction
Ginseng and ginsenosides	Neurotransmitter regulation; mitochondrial and energy metabolism; Wnt2b/GSK‐3*β*/SMAD4; AMPK/Nrf2	A*β* accumulation; tau hyperphosphorylation; neurotransmitter dysfunction; mitochondrial impairment; oxidative stress; neuroinflammation
Danggui Shaoyao San	RAGE/LRP1; PINK1‐Parkin‐mediated mitophagy; AMPK/Sp1/ACSL4; IRS1/GSK3*β*/Wnt3a‐*β*‐catenin; gut microbiota modulation	A*β* clearance disorder; mitochondrial dysfunction; neuronal apoptosis; ferroptosis; glucose metabolism disorder; neuroinflammation; oxidative stress; gut microbiota imbalance
Kai‐Xin‐San	Wnt/*β*‐catenin; SIRT3/NLRP3; SIRT1/FSP1; gut microbiota‐metabolite regulation	A*β* deposition; tau hyperphosphorylation; synaptic dysfunction; neuroinflammation; mitochondrial dysfunction; pyroptosis; ferroptosis; cholinergic dysfunction; gut microbiota‐related changes

**FIGURE 7:**
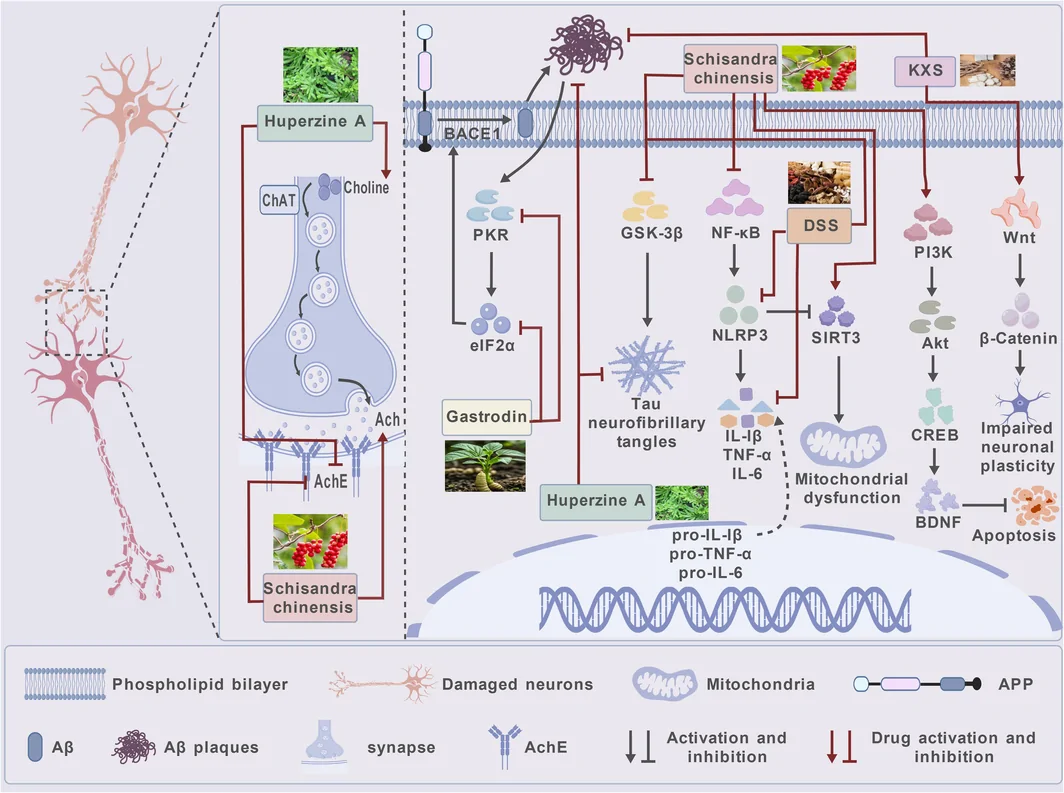
Molecular mechanisms of NMs in treating AD. (Created with BioGDP.com).

### 4.5 Single Compounds

#### 4.5.1 Huperzine A

Huperzine A, a natural alkaloid derived from the club moss *Huperzia serrata* , has been researched since the 1980s. Research interest in AD rose sharply in the early 21st century, with publication output peaking in 2022. As of April 2025, 72 Huperzine A‐related publications were identified in the bibliometric dataset, reflecting sustained research interest in its potential role in AD.

Huperzine A is a highly effective and reversible acetylcholinesterase inhibitor, significantly increasing acetylcholine levels in the brain and improving cholinergic neurotransmission (Zhang and Tang 2006). Beyond its cholinergic mechanism, Huperzine A exhibits multiple neuroprotective effects, including inhibition of *β*‐amyloid aggregation and toxicity, reduction of tau protein hyperphosphorylation, and suppression of neuronal apoptosis (Wang and Tang 2005). Furthermore, Huperzine A improves mitochondrial function, reduces A*β*‐induced mitochondrial damage, and inhibits apoptosis by downregulating p53 protein expression (Tao et al. 2013; Yang et al. 2012).

Huperzine A has also been evaluated in clinical studies of AD. Earlier trials and a meta‐analysis have suggested possible benefits for cognitive function and activities of daily living, although the overall evidence should be interpreted cautiously because of limitations in study quality (Yang et al. 2013). In a multicenter Phase II trial, the prespecified primary efficacy endpoint was not met at the 200 μg twice‐daily dose, while the 400 μg twice‐daily dose showed a cognitive signal only in secondary analyses (Rafii et al. 2011). The clinical evidence for Huperzine A is discussed further in the section on clinical translation.

To address the shortage of natural resources, researchers have successfully isolated Huperzine A‐producing endophytic fungal strains from *H. serrata* (Su and Yang 2015). Additionally, novel drug delivery systems such as nanostructured lipid carriers and nasal temperature‐ and pH‐responsive in situ gels have been developed to enhance bioavailability (Chen et al. 2019). Further well‐designed clinical studies are still needed to clarify the therapeutic value and clinical applicability of Huperzine A in AD.

#### 4.5.2 Quercetin

Quercetin was one of the most frequently occurring single compounds in the bibliometric results (Table S4). Experimental studies suggest that its effects in AD are closely related to A*β* pathology and oxidative stress. Quercetin inhibited A*β* fibril formation and promoted the disaggregation of preformed fibrils in vitro. In APPswe cells, it also reduced ROS production, increased intracellular glutathione, and decreased lipid peroxidation (Jiménez‐Aliaga et al. 2011). Evidence from APP/PS1 mice further showed that dietary quercetin given during the early‐to‐middle stage of disease development improved cognitive performance, accompanied by increased A*β* clearance and reduced astrogliosis; the same benefit was less evident when treatment was initiated at a later stage (Lu et al. 2018). More recently, quercetin was found to reduce the astrocytic uptake of pyroglutamylated A*β*3(pE)‐42 and attenuate the associated astrogliosis and synaptic dysfunction (Arndt et al. 2025). These findings suggest that quercetin may act at several points in A*β*‐related pathology, including aggregation, clearance, oxidative injury, and glial responses.

#### 4.5.3 Curcumin

Curcumin, a major polyphenolic constituent of *Curcuma longa* , has been extensively studied in experimental models of AD. In Tg2576 mice, dietary curcumin reduced oxidative protein damage and IL‐1*β* levels and was associated with lower soluble and insoluble A*β* levels and plaque burden (Lim et al. 2001). Autophagy appears to be another mechanism involved in its effects. In APP/PS1 mice, curcumin improved spatial learning and reduced A*β* accumulation while increasing autophagic activity, together with reduced PI3K, phosphorylated Akt, and mTOR signaling (Wang et al. 2014). Further work in N2a/APP695swe cells showed that curcumin promoted autophagosome formation and autophagosome–lysosome fusion and enhanced the expression of proteins involved in retrograde axonal transport, thereby improving autophagic flux (Liang et al. 2019). Thus, the available experimental evidence links curcumin mainly to the regulation of A*β* pathology, oxidative and inflammatory injury, and impaired autophagic clearance in AD.

#### 4.5.4 Resveratrol

Resveratrol has also been repeatedly investigated for its neuroprotective effects in A*β*‐related models. Subsequent work provided a more specific mechanistic link between resveratrol and SIRT1 signaling. In A*β*25–35‐treated PC12 cells, resveratrol reduced apoptosis and restored intracellular Ca^2+^ homeostasis, while activation of SIRT1 was accompanied by downregulation of ROCK1 (Feng et al. 2013). Resveratrol also promoted mitophagy in A*β*1–42‐treated PC12 cells, increasing markers including Parkin, Beclin‐1, and the LC3‐II/LC3‐I ratio. Inhibition of autophagy weakened its effects on oxidative stress, apoptosis, and mitochondrial damage, indicating that mitochondrial quality control contributes to its neuroprotective action (Wang, Jiang, et al. 2018). These studies mainly support a role for resveratrol in regulating oxidative stress, mitochondrial dysfunction, and A*β*‐induced neuronal injury.

### 4.6 Medicinal Herbs

#### 4.6.1 Schisandra Chinensis

Schisandra chinensis is a commonly used TCM. Recent years have witnessed a steady rise in research reports on its anti‐AD effects, with annual publications peaking at over five papers. Extracts of Schisandra chinensis and its active components (such as schisandrin, schisantherin A, schisantherin B, schisandrin C, schisanhenol, and Schisandra polysaccharides) have demonstrated significant neuroprotective effects (Qi et al. 2019), along with various pharmacological activities including anti‐inflammatory, antioxidant, and anti‐apoptotic properties (Fu et al. 2023).

The mechanisms of action of Schisandra chinensis involve multiple aspects. It can increase acetylcholine levels, inhibit acetylcholinesterase activity, and activate M1 receptors (Wang, Jiang, et al. 2018; Wang Bi, et al. 2018). Regarding the inhibition of A*β* deposition and tau protein hyperphosphorylation, its mechanisms involve the modulation of signaling pathways such as GSK‐3*β* and PI3K/Akt/CREB (Qi et al. 2019). In terms of anti‐inflammatory and antioxidant effects, Schisandra chinensis can inhibit the activation of the NF‐κB pathway and the NLRP3 inflammasome (Qi et al. 2019), and enhance the activity of antioxidant enzymes such as superoxide dismutase (SOD) and glutathione peroxidase (GSH‐Px) (Mao et al. 2015). In the regulation of gut microbiota and metabolism, the Schisandra polysaccharide SCP2 ameliorates metabolic disorders and neuroinflammation in AD model rats by restoring gut microbiota balance and increasing short‐chain fatty acids (Fu et al. 2023). As to the activation of neuroprotective pathways, Schisandra chinensis activates the SIRT1‐PGC‐1*α*‐Tau pathway (Han et al. 2019) and exhibits an activating effect on the BDNF/ERK/CREB pathway (Park et al. 2019). In terms of compatibility and application, Schisandra chinensis exhibits synergistic neuroprotective effects when combined with Acorus tatarinowii Schott, contributing to improvements in cognitive function and modulation of metabolic profiles in Alzheimer's disease (Chen, Hao, et al. 2022; Chen, Huang, et al. 2022).

#### 4.6.2 Gastrodia Elata Blume

Gastrodia elata Blume, a well‐known TCM, has become a prominent focus of research for the treatment of neurological disorders due to its significant neuroprotective effects. Research in this field has remained highly active from 2018 to 2024, yielding a substantial body of high‐quality studies. These investigations have revealed the therapeutic potential and underlying mechanisms of Gastrodia elata and its active components in various neurological conditions, including AD, cerebral ischemia, Parkinson's disease (PD), and epilepsy.

Gastrodin, a glycoside, is the most extensively studied active constituent of Gastrodia elata. It possesses multiple pharmacological activities, including antioxidant, anti‐inflammatory, inhibition of A*β*‐induced toxicity, and modulation of neurotransmitters. Its mechanisms of action are primarily associated with the activation of the Nrf2/HO‐1 antioxidant pathway (Luo et al. 2018). Furthermore, gastrodin elicits its therapeutic effects through the inhibition of the PKR/eIF2*α* signaling pathway, thereby reducing BACE1 expression. (Zhang et al. 2016), and is involved in regulating aging‐ and stress‐related pathways such as DAF‐16/FOXO (Liu et al. 2018). 4,4′‐Methylenediphenol is another key active ingredient. In a *C. elegans* AD model, it significantly delayed A*β*‐induced paralysis and reduced protein deposition by activating the DAF‐16 pathway (Yu et al. 2024).

Gastrodin has demonstrated robust neuroprotective effects in cerebral ischemia models. It can reduce infarct volume and promote neurological functional recovery by upregulating Nrf2 and inhibiting Zn^2+^‐induced toxicity (Luo et al. 2018). Furthermore, the crude extract of Gastrodia elata has been demonstrated in an AD rat model to improve spatial memory, reduce amyloid plaque deposition in the hippocampus, and positively modulate cholinergic system function (Huang et al. 2013).

#### 4.6.3 Ginseng and Ginsenosides

Ginseng was another high‐frequency medicinal herb identified in the bibliometric analysis (Table S4), while much of its mechanistic evidence in AD has focused on its major active constituents, ginsenosides. In a D‐galactose/AlCl3‐induced AD rat model, ginsenoside treatment improved spatial memory, reduced A*β* and phosphorylated tau levels, and partially restored abnormalities in several neurotransmitter systems, including acetylcholine, GABA, dopamine, glutamate, and aspartate (Zhang, Pi, et al. 2016). Individual ginsenosides appear to act through somewhat different mechanisms. Ginsenoside Rg3 improved cognitive impairment in an AD rat model and was associated with improved mitochondrial function, energy metabolism, and reduced apoptosis (Zhang, Yang, et al. 2019). Ginsenoside Rh4 reduced A*β* accumulation and tau hyperphosphorylation, suppressed microglial and astrocytic activation, and regulated the Wnt2b/GSK‐3*β*/SMAD4 pathway (Ren et al. 2023). In APP/PS1 mice, ginsenoside Rk3 improved learning and memory, reduced oxidative stress and glial activation, and activated AMPK/Nrf2 signaling (She et al. 2023). Together, these studies indicate that the effects reported for ginseng cannot be attributed to a single pathway; different ginsenosides have been linked to neurotransmitter dysfunction, A*β* and tau pathology, mitochondrial impairment, oxidative stress, and neuroinflammation.

### 4.7 Chinese Herbal Formulae Used in AD

#### 4.7.1 Danggui Shaoyao San

Danggui Shaoyao San (DSS) is a classic TCM formula. Investigations into its application for AD were initiated in 2005, with a peak in annual publications occurring between 2021 and 2024, averaging over 3 papers per year. DSS possesses various neuroprotective effects, including improving cognitive function, reducing neuronal damage, and inhibiting amyloid deposition (Yang et al. 2021). Its mechanisms of action include downregulating the receptor for advanced glycation end products and upregulating low‐density lipoprotein receptor‐related protein‐1 to promote the clearance of *β*‐amyloid (Yang et al. 2021).

DSS can activate the PINK1‐Parkin‐mediated mitophagy pathway, enhance mitochondrial quality control, reduce neuronal apoptosis, and improve learning and memory abilities in A*β*1‐42‐induced AD rats (Song et al. 2021). Additionally, DSS modulates the gut microbial composition in AD mice, increases the abundance of beneficial bacteria, restores microbial diversity, and exerts therapeutic effects via the gut‐brain axis (Liu et al. 2025).

Recently, research found that DSS inhibited neuronal ferroptosis through the AMPK/Sp1/ACSL4 signaling pathway, alleviated lipid peroxidation damage, and improved cognitive dysfunction (Gong et al. 2025). Metabolomic studies revealed that DSS also improved cerebral glucose metabolism via the IRS1/GSK3*β*/Wnt3a‐*β*‐catenin pathway, alleviating energy metabolism disorders associated with AD (Zhang et al. 2024). Simultaneously, DSS reduced the expression levels of pro‐inflammatory cytokines IL‐1*β*, IL‐6, and TNF‐*α* in the hippocampus and enhanced the activity of antioxidant enzymes such as superoxide dismutase and catalase (He et al. 2023).

To date, various active components such as polysaccharides, phthalides, and flavonoids have been identified in DSS, but the specific effective component groups and their synergistic mechanisms require further clarification.

#### 4.7.2 Kai‐Xin‐San

Kai‐Xin‐San (KXS) is a widely used TCM formula initially recorded in Beiji Qianjin Yaofang since the Tang Dynasty. Modern research on its mechanisms in AD began to emerge around 2020 and reached a peak in research activity in 2024. Kai‐Xin‐San exerts multi‐target neuroprotective effects, including inhibiting A*β* deposition, reducing tau protein hyperphosphorylation, regulating the cholinergic system, and countering neuroinflammation (Luo et al. 2020). Its mechanisms of action also involve ameliorating synaptic plasticity and promoting neuronal survival by activating the Wnt/*β*‐catenin signaling pathway (Shan et al. 2024).

In vitro cell experiments showed that A*β* induction leads to mitochondrial dysfunction and activates the NLRP3 inflammasome in HT22 cells; serum containing Kai‐Xin‐San could upregulate SIRT3 protein expression, thereby inhibiting NLRP3 inflammasome activation and mitigating mitochondrial damage and pyroptosis (Su et al. 2023). Kai‐Xin‐San inhibits neuronal ferroptosis in the hippocampus of APP/PS1 transgenic mice and improves their learning and memory deficits by activating the SIRT1/FSP1 signaling pathway (Yan et al. 2024). Several active components have been identified in Kai‐Xin‐San, including ginsenosides (e.g., Rg1, Rb1), polygala saponins (e.g., polygalaxanthone III), and Acorus volatile oils (e.g., *β*‐asarone) (Wu et al. 2025; Zhang et al. 2015). Future research needs to delve deeper into its mechanism of action via the “gut microbiota‐metabolite‐brain axis” (Cai et al. 2025) and promote formulation standardization and precise evaluation of clinical efficacy.

### 4.8 A Summary of the Inherent Molecular Mechanisms

The core pathological processes of AD include *β*‐amyloid (A*β*) deposition, hyperphosphorylation of Tau protein, neuroinflammation, oxidative stress, and synaptic dysfunction, among others. Based on this, modern treatment strategies, particularly TCM, demonstrate characteristics of multi‐target interventions. The main molecular mechanisms include: inhibiting A*β* production and aggregation, reducing Tau protein phosphorylation, suppressing neuroinflammation, ameliorating oxidative stress, and regulating autophagy and mitochondrial function (Table 6).

**TABLE 6:** Molecular mechanisms, experimental models, and key effects of NMs/formulas against AD.

Molecular mechanism	Natural medicine/formula	Experimental model	Key effects
A*β* deposits	Ginsenoside Rg1	Primary cultured rat hippocampal neurons induced by A*β*_1‐42_	Accelerate the degradation of a*β*
	Huanglian Jiedu Decoction	Tg‐APP/PS1 mice	Modulating the gut microbiota, intervening in a*β* pathology
Tau neurofibrillary tangles	Danggui Shaoyao San	APP/PS1 mice	Regulation of the gsk3*β*/pgc1*α* signalling pathway
	Huperzine A	SH‐SY5Y neuroblastoma cells	Inhibiting gsk‐3*β* activity
	Icariin	3 × Tg‐AD transgenic mouse model	Inhibiting gsk‐3*β* through the sirt1 signaling pathway
Cholinergic dysfunction	Cornuside	Scopolamine induced AD mice	Regulating ach levels
Neuroinflammation	Astragaloside IV	LPS induced BV2 cell model	Pro‐inflammatory m1 phenotype
Oxidative stress	Astaxanthin	Scopolamine induced AD rat	Activate the nrf2 pathway, enhance the expression of downstream antioxidant enzymes

### 4.9 A*β* Deposits and Tau Neurofibrillary Tangles

The A*β* cascade hypothesis is one of the core pathological mechanisms of AD. Interventions with natural medicines are reflected not only in inhibiting the activity of *β*‐secretase (BACE1) and γ‐secretase to reduce A*β* production but also in promoting the clearance of A*β*. For instance, in a primary cultured rat hippocampal neuron cell model induced by A*β*_1‐42_, ginsenoside Rg1 can accelerate the degradation of A*β* by regulating the expression of insulin‐degrading enzyme (IDE), thereby reducing plaque deposition in the brain (Quan et al. 2024). In Tg‐APP/PS1 mice, Huanglian Jiedu Decoction has been proven to indirectly affect systemic and central nervous system inflammation by modulating the gut microbiota, thereby intervening in A*β* pathology (Gu et al. 2021) (Figure 8A).

**FIGURE 8:**
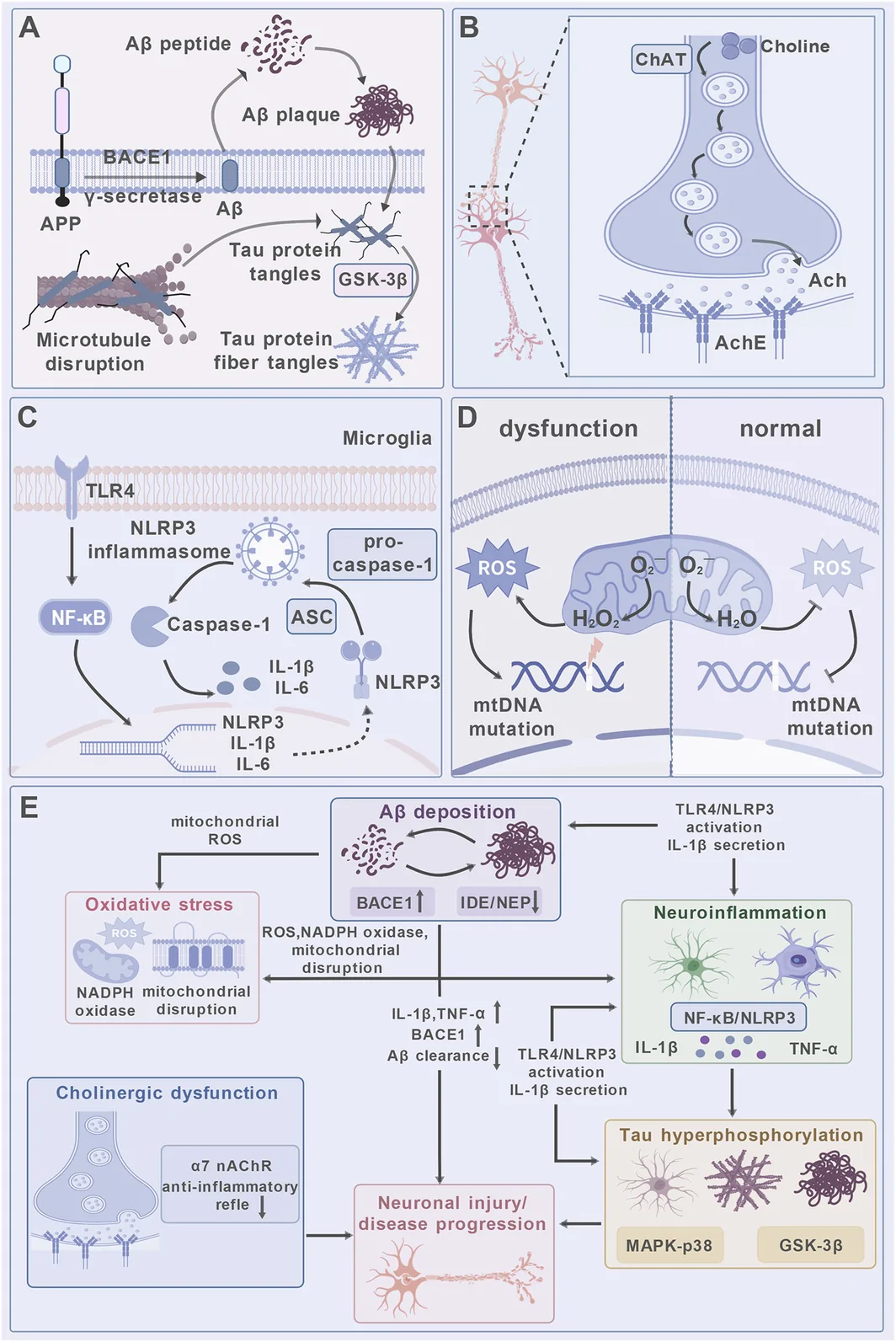
The pathogenesis of AD. (A) A*β* deposits and tau neurofibrillary tangles. (B) Cholinergic dysfunction. (C) Neuroinflammation. (D) Oxidative stress. (E) Interplay among multiple pathological mechanisms driving AD progression. (Created with BioGDP.com).

Hyperphosphorylation of Tau protein leading to neurofibrillary tangles is another major characteristic of AD. Glycogen synthase kinase‐3*β* (GSK‐3*β*) is a key kinase regulating Tau phosphorylation. Through comprehensive multi omics methods, it was found that DSS improves cognitive impairment in APP/PS1 mice by regulating the GSK3*β*/PGC1 *α* signaling pathway (Wu et al. 2024). In SH‐SY5Y neuroblastoma cells, it was demonstrated that huperzine A reduces GSK3*β* protein levels and enhances the inhibitory phosphorylation of GSK3*β* at Ser9 (the inactivated form), accompanied by a marked reduction in tau protein phosphorylation (Wongjaikam et al. 2024). In the 3 × Tg‐AD transgenic mouse model, icariin was reported to inhibit microglial and astrocytic activation, reduce inflammatory cytokines including IL‐1*β*, TNF‐*α*, and IL‐6, and modulate the Akt/GSK‐3*β* signaling pathway, thereby alleviating AD‐related pathological changes (Wu et al. 2024) (Figure 8A).

### 4.10 Cholinergic Dysfunction

The cholinergic system plays a central role in the pathogenesis of AD, primarily due to the significant degenerative loss of cholinergic neurons in the basal forebrain. This leads to a dramatic decline in acetylcholine (ACh) levels in critical brain regions such as the cerebral cortex and hippocampus. ACh is a neurotransmitter essential for learning, memory, and attention, and its deficiency directly causes cognitive impairment. Moreover, the cholinergic hypothesis not only represents one of the earliest proposed mechanisms for AD but also underpins the continued use of acetylcholinesterase inhibitors (such as donepezil) as the standard first‐line therapeutic strategy for symptomatic improvement of cognitive function in AD patients (Chen, Huang, et al. 2022; Ferreira‐Vieira et al. 2016). In scopolamine induced AD mice, cornuside has been demonstrated to improve cholinergic dysfunction by regulating ACh levels (Wang et al. 2022) (Figure 8B).

### 4.11 Neuroinflammation

Neuroinflammation is critical to the pathological process underlying AD, with overactivation of microglia being a key feature. By inhibiting the Toll‐like receptor 4 (TLR4) signaling pathway, it effectively reduces the activation of the NLRP3 inflammasome and the release of pro‐inflammatory factors (Li et al. 2024). In the LPS‐induced BV2 cell model, astragaloside IV has also been shown to inhibit the polarization of microglia toward the pro‐inflammatory M1 phenotype by regulating this pathway, thereby alleviating neuroinflammatory damage (He, Sun, et al. 2023) (Figure 8C).

### 4.12 Oxidative Stress

Oxidative stress is closely associated with mitochondrial dysfunction and neuronal death in AD. The core mechanism involves an imbalance between excessive reactive oxygen species (ROS) production and clearance. When ROS levels exceed a cell's antioxidant defense capacity, they can damage neuronal components and contribute to neuronal death. Excessive oxidative stress can also impair mitochondrial function and mitochondrial homeostasis, further aggravating neuronal injury (Bartman et al. 2024) (Figure 8D).

Through the above multiple mechanisms, natural medicines form a synergistic network that enables multi‐dimensional and systematic intervention in AD, demonstrating significant therapeutic potential.

### 4.13 Interplay Among Multiple Pathological Mechanisms

While A*β* deposition, Tau hyperphosphorylation, cholinergic dysfunction, neuroinflammation, and oxidative stress are often described as discrete pathological processes in AD, a growing body of evidence indicates that these mechanisms are not independent but rather engage in complex, bidirectional interactions that collectively drive disease progression. Understanding these interconnections is essential for developing multi‐target therapeutic strategies, particularly NMs that simultaneously modulate multiple pathways.

Oxidative stress exacerbates neuroinflammation, which in turn promotes A*β* deposition and Tau pathology. ROS activate NF‐κB, triggering pro‐inflammatory cytokine release (IL‐1*β*, TNF‐*α*) from microglia and astrocytes. These cytokines further stimulate ROS production via NADPH oxidase and mitochondrial disruption, forming a self‐amplifying cycle. Notably, IL‐1*β* and TNF‐*α* upregulate BACE1 (increasing A*β* generation) and downregulate IDE/NEP (impairing A*β* clearance). IL‐1*β* also drives tau hyperphosphorylation via MAPK‐p38 activation. Conversely, A*β* aggregates induce mitochondrial ROS and act as DAMPs binding to TLR4 on microglia, activating NLRP3 inflammasome and IL‐1*β* secretion. These changes further promote A*β* aggregation in a feed‐forward loop (Latz 2010; Peng et al. 2024). Additionally, cholinergic dysfunction worsens neuroinflammation by disrupting the *α*7 nAChR‐mediated anti‐inflammatory reflex, while *α*7 nAChR activation antagonizes A*β*‐induced inflammation (Benfante et al. 2021; Rennie 2025).

Taken together, these interactions form a highly integrated and self‐perpetuating pathological network. This also explains why the efficacy of single target therapy in AD is often limited and supports the theoretical basis of NMs targeting multiple targets simultaneously (Figure 8E).

### 4.14 Computational Approaches in Natural Medicine Research for Alzheimer's Disease: Applications and Methodological Considerations

Beyond the identification of representative natural medicines, formulae, and mechanistic themes, the bibliometric analysis also revealed the increasing representation of methodological approaches used to investigate these complex multi‐component systems. Among these approaches, network pharmacology and molecular docking emerged as important methodology‐related themes in natural medicine research for AD. These computational approaches have been incorporated into natural medicine studies to organize candidate compounds, predict potential targets and pathways, and prioritize compound–target interactions for further investigation.

Representative studies have applied network pharmacology and molecular docking to investigate potential mechanisms of natural medicines in AD. For example, these approaches have been used to explore the potential mechanisms of Kai‐Xin‐San (Yi et al. 2020), Huanglian Jiedu Decoction (Zheng et al. 2023), and curcumin (Wu et al. 2022). Similar strategies have also been applied to medicinal herbs, including *Acanthopanax senticosus* (Zhuo et al. 2023), Polygonatum sibiricum (Luo et al. 2024), and Acorus tatarinowii Rhizoma (Tong et al. 2024). Some studies have further extended computational analyses by incorporating experimental validation. For instance, Zhuo et al. combined chemical profiling, network analysis, and experimental approaches to investigate the effects of *Acanthopanax senticosus* in AD‐related models (Zhuo et al. 2023).

Despite their usefulness for hypothesis generation, network pharmacology and molecular docking have important methodological limitations. Network pharmacology analyses depend heavily on the databases and prediction algorithms used for compound and target identification. Comparisons of commonly used TCM databases have demonstrated differences in compound coverage, compound–target associations, and data redundancy, which may result in different candidate targets being identified for the same herb depending on the database and analytical workflow (Zhang, Zhu, et al. 2019). In addition, target prediction strategies and screening thresholds vary substantially among studies, potentially affecting downstream protein–protein interaction networks, enrichment analyses, and the selection of highly connected targets. Therefore, highly ranked hub targets should not be interpreted as direct evidence of causal involvement in pharmacological effects without experimental validation.

Molecular docking provides additional information regarding potential compound–target interactions but also has important interpretive limitations. Docking can predict plausible binding poses and assist in ranking candidate interactions; however, docking scores do not directly demonstrate target binding, biological activity, or therapeutic efficacy. The reliability of docking results can be influenced by factors including protein and ligand preparation, conformational flexibility, scoring functions, and solvent effects (Torres et al. 2019). Therefore, favorable docking results should be considered supportive computational evidence for hypothesis generation rather than confirmation of target engagement or biological mechanisms.

Overall, network pharmacology and molecular docking should primarily be regarded as tools for generating and prioritizing mechanistic hypotheses. Predictions regarding candidate compounds, targets, and pathways require further validation through complementary approaches, including biochemical or biophysical binding assays, genetic or pharmacological perturbation, cellular functional studies, pharmacokinetic evaluation, and in vivo experiments. The increasing number of computational studies should not be interpreted as equivalent to an increase in experimentally established mechanisms, particularly when investigations remain limited to prediction or docking analyses. Integrating computational approaches with chemical characterization, multi‐omics analyses, and experimental validation may provide a more reliable strategy for understanding the complex pharmacology of natural medicines in AD.

### 4.15 Clinical Translation Status and Major Challenges

Clinical evidence for NMs does not necessarily parallel their prominence in bibliometric or mechanistic research. Therefore, this section was not restricted to the medicines selected for mechanistic discussion but also considered NMs for which human evidence is relatively well developed.

Among these, standardized *Ginkgo biloba* extract EGb 761 has been evaluated in several randomized, placebo‐controlled trials in patients with dementia. A 24‐week multicenter trial involving 410 patients with mild‐to‐moderate dementia showed that EGb 761 at 240 mg/day improved cognitive performance, neuropsychiatric symptoms, and functional outcomes compared with placebo (Herrschaft et al. 2012). A subsequent systematic review and meta‐analysis also found that the 240 mg/day dose was associated with benefits in cognition, function, behavior, and global assessment, particularly in patients with neuropsychiatric symptoms (Tan et al. 2015). These studies place EGb 761 among the more extensively evaluated natural medicine preparations in dementia.

Huperzine A has also been investigated in randomized trials and systematic reviews in AD. In a multicenter Phase II trial involving 210 patients with mild‐to‐moderate AD, the 200 μg twice‐daily dose did not improve the prespecified primary cognitive endpoint at week 16. The 400 μg twice‐daily dose showed an improvement in ADAS‐Cog in a secondary analysis at week 11, although the difference was no longer statistically significant at week 16 (Rafii et al. 2011). Earlier trials and a subsequent systematic review reported possible improvements in cognitive function and activities of daily living, but these findings should be interpreted cautiously because of limitations in the methodological quality of the available studies (Yang et al. 2013).

Resveratrol has progressed from extensive preclinical investigation to early clinical evaluation in AD. In a 52‐week randomized, double‐blind, placebo‐controlled Phase II trial involving 119 patients with mild‐to‐moderate AD, resveratrol was generally safe and well tolerated and was detectable in both plasma and cerebrospinal fluid. Treatment was associated with changes in several AD‐related biomarkers, although the study did not establish a clear clinical cognitive benefit (Turner et al. 2015). Thus, the available evidence supports further clinical investigation rather than a definitive therapeutic effect.

Clinical evidence for Gastrodia elata remains less mature. A 12‐week randomized, double‐blind, placebo‐controlled study involving 100 middle‐aged adults with normal cognition or mild cognitive impairment reported improvements in cognitive measures and several biochemical outcomes, including K‐MMSE, BDNF, and total antioxidant status, without serious adverse events (Jun et al. 2025). However, the study was not conducted in patients with established AD, and its relatively small sample size and short duration limit the conclusions that can be drawn regarding AD treatment.

Human studies have also been reported for several other NMs, although the evidence remains limited. A pilot randomized, double‐blind, placebo‐controlled trial evaluated curcumin in patients with AD (Baum et al. 2008), while small clinical studies of *Panax ginseng* have reported possible improvements in cognitive performance in patients with AD (Lee et al. 2008). However, the available evidence for ginseng remains scarce and inconclusive (Lee et al. 2009). Danggui Shaoyao San has also been evaluated in randomized trials included in a systematic review and meta‐analysis of dementia, but the available studies were limited by small sample sizes and methodological weaknesses (Kim and Cho 2020). In contrast, robust AD‐specific clinical evidence remains sparse for several other medicines discussed in the mechanistic section, including Schisandra chinensis and Kai‐Xin‐San.

Together, these findings show a marked difference in the maturity of clinical evidence across NMs. Extensive mechanistic or preclinical research does not necessarily translate into an equivalent level of clinical evidence. The current clinical evidence of representative NMs is summarized in Table 7.

**TABLE 7:** Clinical evidence and translational status of representative natural medicines for cognitive impairment and Alzheimer's disease.

Natural medicine	Representative clinical evidence	Study population	Sample size/duration	Main findings
EGb 761	Randomized, placebo‐controlled trials; systematic reviews/meta‐analyses	Mild‐to‐moderate dementia, including AD and mixed dementia	*n* = 410; 24 weeks	At 240 mg/day, improvements were reported in cognition, neuropsychiatric symptoms, and functional outcomes; meta‐analyses also suggested cognitive and functional benefits.
Huperzine A	Multicenter Phase II randomized, double‐blind, placebo‐controlled trial; additional RCTs and meta‐analysis	Mild‐to‐moderate AD	*n* = 210; 16 weeks	The 200 μg twice‐daily dose did not meet the prespecified primary cognitive endpoint. The 400 μg twice‐daily dose showed improvement only in secondary analysis.
Resveratrol	Randomized, double‐blind, placebo‐controlled Phase II trial	Mild‐to‐moderate AD	*n* = 119; 52 weeks	Generally safe and well tolerated; biomarker changes were observed, but clear cognitive efficacy was not established.
Gastrodia elata	Randomized, double‐blind, placebo‐controlled clinical study	Adults with normal cognition or mild cognitive impairment	*n* = 100; 12 weeks	Improvements were reported in cognitive measures and biomarkers; AD‐specific therapeutic efficacy remains unclear.
Curcumin	Randomized, double‐blind, placebo‐controlled pilot trial	Patients with AD	*n* = 34; 6 months	No significant cognitive improvement was demonstrated in this pilot study.
*Panax ginseng*	Prospective open‐label clinical study	Patients with AD	58 ginseng‐treated and 39 control participants; 12 weeks	Cognitive improvement was reported, but evidence remains limited by study design.
Danggui Shaoyao San	Clinical studies summarized in a systematic review and meta‐analysis	Patients with dementia	5 RCTs	Potential cognitive and functional benefits were suggested, although conclusions are limited by study quality.
Schisandra chinensis	No robust AD‐specific randomized clinical evidence identified	—	—	Clinical efficacy in established AD has not been demonstrated in controlled trials.
Kai‐Xin‐San	Clinical evidence remains limited; current evidence is mainly preclinical	—	—	Preclinical studies report cognitive and mechanistic effects, but clinical efficacy in established AD remains unconfirmed.

Beyond the differences in clinical evidence among individual medicines, several broader issues continue to limit translation. Many natural products have poor or variable bioavailability, while herbal extracts and formulae can show substantial variation in composition depending on plant source, processing, and manufacturing procedures. These factors complicate dose standardization and reproducibility across studies. In addition, many clinical studies have involved relatively small samples, short treatment periods, and heterogeneous patient populations. Future trials will require better characterized preparations, pharmacokinetic evaluation, longer follow‐up, and clinically relevant biomarker and cognitive endpoints.

### 4.16 Limitations of the Present Study

The mechanistic section of this study should also be interpreted with some caution. It was designed as a focused narrative synthesis guided by the main themes identified in the bibliometric analysis rather than as an exhaustive systematic review. Therefore, the experimental studies discussed in this section do not represent all available evidence, and no formal risk‐of‐bias assessment was performed. The mechanistic findings are intended mainly to place the bibliometric results in the context of existing experimental evidence and to highlight areas that warrant further investigation, rather than to compare the efficacy of individual natural medicines or support clinical recommendations.

## 5 Conclusions and Perspectives

This study mapped the development, knowledge structure, and major research themes of natural medicine research for AD from January 1, 2001, to April 28, 2025 by combining bibliometric analysis with a focused narrative synthesis. The bibliometric findings showed that research in this field has gradually shifted from early studies of cholinesterase inhibition and antioxidant effects toward broader investigations of A*β* and tau pathology, neuroinflammation, oxidative stress, and other AD‐related mechanisms. The subsequent review of experimental studies provided further context for these themes and summarized the mechanisms reported for representative natural medicines.

The available evidence also shows considerable differences in the maturity of research across individual medicines. Some agents, such as EGb 761 and Huperzine A, have progressed further into clinical investigation, whereas many other natural medicines remain supported mainly by preclinical studies. Gut microbiota‐related research has attracted increasing attention in recent years, but it remains an emerging direction and is still based largely on experimental models. These findings highlight the gap that remains between mechanistic research and clinical translation.

Computational approaches such as network pharmacology and molecular docking are frequently applied in this field and may assist in identifying candidate compounds, targets, and pathways. However, these approaches should primarily serve as hypothesis‐generating tools rather than direct evidence of target engagement or therapeutic activity. Future studies should place greater emphasis on experimental validation of computational predictions and integrate chemical characterization, multi‐omics approaches, pharmacokinetic evaluation, and well‐designed cellular and animal studies.

Further clinical development will also require better standardization of natural medicine preparations, clearer characterization of active constituents and pharmacokinetic properties, and adequately powered clinical trials with appropriate biomarkers and clinically meaningful outcomes. Greater attention to these issues will be important for determining which mechanistic findings can ultimately be translated into clinically relevant interventions for AD.

Overall, this study provides an overview of how natural medicine research in AD has developed and identifies several areas in which stronger experimental and clinical evidence is still needed.

## Author Contributions

**Yunhui Hu:** writing – review and editing. **Qian Zhao:** writing – review and editing. **Wenjia Wang:** writing – review and editing. **Maoning Wang:** writing – original draft, methodology, visualization. **Jianhe Zhao:** investigation, formal analysis. **Xiaolian Zhang:** writing – original draft, visualization. **Huaxian Yang:** investigation, formal analysis, data curation. **Jiansong Fang:** writing – review and editing, funding acquisition, conceptualization. **Wentao Wei:** writing – review and editing.

## Funding

This work was supported by National Key Research and Development Program of China, 2023YFF1204901, Basic and Applied Basic Research Foundation of Guangdong Province, 2025A1515012991, Special Foundation of Guangdong Educational Committee, 2025ZDZX2006.

## Conflicts of Interest

The authors declare no conflicts of interest.

## Supporting information


**Table S1:** The top 10 author co‐occurrence regarding natural medicines against AD from January 1, 2001, to April 28, 2025.
**Table S2:** The top 10 country co‐occurrence regarding natural medicines against AD from January 1, 2001, to April 28, 2025.
**Table S3:** The top 10 author co‐citation regarding natural medicines against AD from January 1, 2001, to April 28, 2025.
**Table S4:** Representative single compounds, herbal medicines, and formulae from the “Traditional Chinese medicine” cluster.
**Table S5:** Complete list of abbreviations used in the manuscript.

## Data Availability

The data that supports the findings of this study are available in the Supporting Information of this article.
