# Supplementary material for: Research Trends and Mechanistic Insights Into Natural Medicines for Alzheimer's Disease: A Bibliometric Analysis and Narrative Review
Source: Phytother Res. 2026 Sep 11;40(11):6490–515. doi: 10.1002/ptr.70445 (PMC13753705; doi:10.1002/ptr.70445)
Supplement: Supplementary file 1 [file PTR-40-6490-s001.docx]

**Supplementary Material**

**Research Trends and Mechanistic Insights into Natural Medicines for Alzheimer's Disease: A Bibliometric Analysis and Narrative Review**

# Maoning Wang^#a^, Xiaolian Zhang^#a^, Huaxian Yang^a^, Jianhe Zhao^a^, Wentao Wei^a^, Qian Zhao^b^, Wenjia Wang^b^, Yunhui Hu^b^, Jiansong Fang*^a^

^a^State Key Laboratory of Traditional Chinese Medicine Syndrome, Science and Technology Innovation Center, Guangzhou University of Chinese Medicine, Guangzhou, 510405, China

^b^Tianjin Tasly Digital Intelligence Chinese Medicine Technology Co., Ltd., Tianjin 300410, China

* Corresponding author

E-mail addresses: fangjs@gzucm.edu.cn (J. Fang).

^#^Maoning Wang and Xiaolian Zhang contributed equally to this work.

**Table S1. The top 10 author co-occurrence regarding natural medicines against AD from January 1, 2001, to April 28, 2025.**

| Author | Documents | Citations | Total link strength | Country |
| --- | --- | --- | --- | --- |
| zengin, gokhan | 51 | 1488 | 48 | Turkey |
| shi, jing | 15 | 198 | 34 | China |
| bi, kaishun | 16 | 528 | 33 | China |
| tian, jinzhou | 12 | 188 | 33 | China |
| jia, ying | 13 | 474 | 32 | China |
| wang, yongyan | 10 | 156 | 31 | Germany |
| yan, tingxu | 10 | 327 | 28 | China |
| wei, mingqing | 9 | 111 | 27 | China |
| wu, bo | 9 | 310 | 26 | China |
| mahomoodally, mohamad fawzi | 17 | 400 | 24 | Vietnam |

**Note: TLS (total link strength) represents the sum of the collaboration link strengths between a given institution and all other institutions in the VOSviewer co-authorship network. Higher TLS values indicate stronger overall collaborative connections in this field.**

**Table S2. The top 10 country co-occurrence regarding natural medicines against AD from January 1, 2001, to April 28, 2025.**

| **Country** | **Document** | **Citations** | **Total link strength** |
| --- | --- | --- | --- |
| usa | 279 | 16015 | 281 |
| saudi arabia | 139 | 1795 | 262 |
| peoples r china | 1395 | 30720 | 261 |
| india | 363 | 7166 | 204 |
| turkey | 126 | 3870 | 172 |
| pakistan | 117 | 2242 | 148 |
| italy | 93 | 3027 | 147 |
| egypt | 105 | 1871 | 129 |
| south korea | 247 | 5892 | 94 |
| germany | 67 | 2769 | 89 |

**Note: TLS (total link strength) represents the sum of the collaboration link strengths between a given institution and all other institutions in the VOSviewer co-authorship network. Higher TLS values indicate stronger overall collaborative connections in this field.**

**Table S3. The top 10 author co-citation regarding natural medicines against AD from January 1, 2001, to April 28, 2025.**

| **Author** | **Citations** | **Total link strength** |
| --- | --- | --- |
| ellman, gl | 539 | 1249 |
| selkoe, dj | 289 | 824 |
| butterfield, da | 243 | 728 |
| zhang, l | 192 | 599 |
| howes, mjr | 162 | 584 |
| orhan, i | 153 | 540 |
| wang, r | 107 | 529 |
| mattson, mp | 175 | 525 |
| mukherjee, pk | 164 | 517 |
| wang, y | 175 | 517 |

**Note: TLS (total link strength) represents the sum of the collaboration link strengths between a given institution and all other institutions in the VOSviewer co-authorship network. Higher TLS values indicate stronger overall collaborative connections in this field.**

**Table S4. Representative single compounds, medicinal herbs, and formulae from the “Traditional Chinese medicine” cluster**

| **Single compound** | **Occurrence** | **Formula** | **Occurrence** | **Medicinal herb** | **Occurrence** |
| --- | --- | --- | --- | --- | --- |
| Huperzine A | 72 | Kai-Xin-San | 26 | Schisandra | 83 |
| Quercetin | 55 | Danggui-Shaoyao-San | 24 | Gastrodia | 49 |
| Curcumin | 49 | Huang-Lian-Jie-Du-Tang | 16 | Ginseng | 47 |
| Galantamine | 46 | Yi-Gan-San | 9 | Ginkgo | 44 |
| Gallic acid | 29 | Bushen-Yizhi Formula | 7 | Epimedium | 30 |
| Resveratrol, including trans-resveratrol | 29 | Bushen Tiansui Formula | 6 | Huperzia | 28 |
| Kaempferol | 28 | Qi-Fu-Yin | 5 | Polygala | 26 |
| Rosmarinic acid | 26 | Fuzhisan | 5 | Salvia | 22 |
| Rutin | 23 | Lingguizhugan decoction | 4 | Coptis | 18 |
| Chlorogenic acid | 22 | Huannao Yicong Formula | 4 | Angelica | 15 |

**Note: Occurrence indicates the number of times each term appeared in the bibliometric dataset.**

**Table S5. Complete list of abbreviations used in the manuscript**

| **Abbreviation** | **Full name** | **Abbreviation** | **Full name** |
| --- | --- | --- | --- |
| 3×Tg-AD | Triple-transgenic Alzheimer’s disease mouse model | JCR | Journal Citation Reports |
| ACh | Acetylcholine | K-MMSE | Korean version of the Mini-Mental State Examination |
| AChE | Acetylcholinesterase | KXS | Kai-Xin-San |
| AChEI | Acetylcholinesterase inhibitor | LPS | Lipopolysaccharide |
| ACSL4 | Acyl-CoA synthetase long-chain family member 4 | LRP1 | Low-density lipoprotein receptor-related protein 1 |
| AD | Alzheimer’s disease | MAPK-p38 | p38 mitogen-activated protein kinase |
| ADAS-Cog | Alzheimer’s Disease Assessment Scale-Cognitive Subscale | MMSE | Mini-Mental State Examination |
| ADL | Activities of daily living | mtDNA | Mitochondrial DNA |
| Akt | Protein kinase B | NADPH | Nicotinamide adenine dinucleotide phosphate |
| AMPK | AMP-activated protein kinase | NEP | Neprilysin |
| APP | Amyloid precursor protein | NF-κB | Nuclear factor kappa B |
| APP/PS1 | Amyloid precursor protein/presenilin 1 | NLRP3 | NOD-like receptor family pyrin domain-containing 3 |
| Aβ | Amyloid beta | NMDA | N-Methyl-D-aspartate |
| Aβ1-42 | Amyloid beta 1-42 | NMs | Natural medicines |
| BACE1 | Beta-site amyloid precursor protein-cleaving enzyme 1 | Nrf2 | Nuclear factor erythroid 2-related factor 2 |
| BBB | Blood-brain barrier | PC12 | Rat pheochromocytoma cell line |
| BDNF | Brain-derived neurotrophic factor | PD | Parkinson’s disease |
| BV2 | Murine microglial cell line | PGC-1α | Peroxisome proliferator-activated receptor gamma coactivator 1 alpha |
| CREB | cAMP response element-binding protein | PI3K | Phosphatidylinositol 3-kinase |
| CSF | Cerebrospinal fluid | PINK1 | PTEN-induced kinase 1 |
| DAF-16/FOXO | Dauer formation 16/forkhead box O | PKR | Protein kinase R |
| DAMPs | Damage-associated molecular patterns | RAGE | Receptor for advanced glycation end products |
| DSS | Danggui Shaoyao San | RCTs | Randomized controlled trials |
| DTNB | 5,5′-Dithiobis-(2-nitrobenzoic acid) | ROS | Reactive oxygen species |
| eIF2α | Eukaryotic initiation factor 2 alpha | SAMP8 | Senescence-accelerated mouse prone 8 |
| ERK | Extracellular signal-regulated kinase | SCFAs | Short-chain fatty acids |
| FRAP | Ferric reducing ability of plasma | SH-SY5Y | Human neuroblastoma cell line |
| FSP1 | Ferroptosis suppressor protein 1 | SIRT1 | Sirtuin 1 |
| GSH-Px | Glutathione peroxidase | SIRT3 | Sirtuin 3 |
| GSK-3β | Glycogen synthase kinase 3 beta | SOD | Superoxide dismutase |
| HO-1 | Heme oxygenase 1 | Sp1 | Specificity protein 1 |
| HT22 | Mouse hippocampal neuronal cell line | TCM | Traditional Chinese medicine |
| IDE | Insulin-degrading enzyme | TLR4 | Toll-like receptor 4 |
| IF | Impact factor | TLS | Total link strength |
| IL-1β | Interleukin-1 beta | TNF-α | Tumor necrosis factor alpha |
| IL-6 | Interleukin-6 | WoS | Web of Science |
| IRS1 | Insulin receptor substrate 1 | α7 nAChR | Alpha 7 nicotinic acetylcholine receptor |
